# Physical and functional interaction between SET1/COMPASS complex component CFP-1 and a Sin3S HDAC complex in *C. elegans*

**DOI:** 10.1093/nar/gkz880

**Published:** 2019-10-11

**Authors:** Flore Beurton, Przemyslaw Stempor, Matthieu Caron, Alex Appert, Yan Dong, Ron A-j Chen, David Cluet, Yohann Couté, Marion Herbette, Ni Huang, Hélène Polveche, Martin Spichty, Cécile Bedet, Julie Ahringer, Francesca Palladino

**Affiliations:** 1 Laboratory of Biology and Modeling of the Cell, UMR5239 CNRS/Ecole Normale Supérieure de Lyon, INSERM U1210, UMS 3444 Biosciences Lyon Gerland, Université de Lyon, Lyon, France; 2 The Gurdon Institute and Department of Genetics, University of Cambridge, Cambridge, UK; 3 Grenoble Alpes, CEA, Inserm, BIG-BGE, 38000 Grenoble, France; 4 INSERM UMR 861, I-STEM, 28, Rue Henri Desbruères, 91100 Corbeil-Essonnes, France

## Abstract

The CFP1 CXXC zinc finger protein targets the SET1/COMPASS complex to non-methylated CpG rich promoters to implement tri-methylation of histone H3 Lys4 (H3K4me3). Although H3K4me3 is widely associated with gene expression, the effects of CFP1 loss vary, suggesting additional chromatin factors contribute to context dependent effects. Using a proteomics approach, we identified CFP1 associated proteins and an unexpected direct link between *Caenorhabditis elegans* CFP-1 and an Rpd3/Sin3 small (SIN3S) histone deacetylase complex. Supporting a functional connection, we find that mutants of COMPASS and SIN3 complex components genetically interact and have similar phenotypic defects including misregulation of common genes. CFP-1 directly binds SIN-3 through a region including the conserved PAH1 domain and recruits SIN-3 and the HDA-1/HDAC subunit to H3K4me3 enriched promoters. Our results reveal a novel role for CFP-1 in mediating interaction between SET1/COMPASS and a Sin3S HDAC complex at promoters.

## INTRODUCTION

The CFP1/CXXC zinc finger protein targets the SET1/COMPASS complex to non-methylated CpG rich regions for trimethylation of histone H3 on Lys4 (H3K4me3) (1–5), a modification widely associated with active promoters (6–8). The roles of CFP1 and the SET1/COMPASS complex in gene regulation are unclear. In different systems, loss of individual subunits does not have widespread effects on transcription, with only small subsets of genes affected (1,2,9–14). The effects vary depending on context, consistent with potential interactions with other factors, and proposals that H3K4me3 may promote transcriptional memory and consistency (9,15,16).

In yeast, SET1 acts in a single complex known as COMPASS (complex of proteins associated with Set1) that is responsible for all H3K4 methylation (17,18). In mammals by contrast, six complexes have been isolated defined by the catalytic subunits SET1A, SET1B, MLL1, MLL2, MLL3 and MLL4 (reviewed in (19). The enzymatic activity of SET1/MLL family members is regulated by interactions with additional proteins, including Swd3/WDR5, Swd1/RbBP5, Bre2/ASH2 and Sdc1/hDPY30 that influence the state (mono-, di- or tri) of methylation deposited (20–22). In addition, unique subunits including CFP1 are associated with each complex and contribute to its specificity (3,23–25).

SET1/MLL complexes have non-redundant functions, as demonstrated by the distinct phenotypes and embryonic lethality caused by deletion of individual SET1/MLL genes (26–29). While SET1 proteins are responsible for global H3K4me3 at promoter regions in different organisms (30–34), MLL proteins deposit H3K4 methylation at specific genes or regulatory elements (35–37).

*Caenorhabditis elegans* contains a single homologue of SET1, named SET-2, one MLL-like protein, SET-16, and single homologs of WDR5, ASH2L, DPY30, RbBP5 and CFP1 (32,34,38,39), simplifying functional studies of SET1/MLL regulatory networks. Inactivation of SET-2, WDR-5.1, DPY-30, RbBP5 and CFP-1 has shown that they all contribute to global H3K4 methylation in the germline and soma, and share common functions in somatic and germline development (32,34,38,40–43). To biochemically analyze the complex and identify associated proteins that may contribute to its functional outcome, we immunoprecipitated tagged CFP-1 and WDR-5.1, and identified copurifying proteins by mass spectrometry. In addition to identifying distinct SET-2/SET1 and SET-16/MLL complexes, we found that WDR-5.1 co-immunoprecipitates NSL histone acetyltransferase (HAT) complex subunits, consistent with its presence in multiple chromatin-associated complexes (44–46). Most importantly, we show that CFP-1 physically and functionally interacts with a conserved Rpd3/Sin3 small histone deacetylase complex, SIN3S. Mutants of SET-2/SET1 and SIN3S complex subunits share partly similar phenotypes, and CFP-1 is important for recruitment of both SIN-3 and the HDA-1/HDAC subunit to H3K4me3 enriched promoters. Our results reveal a novel role for CFP-1 in interacting with both SET-2/SET1 and SIN3S HDAC complexes to maintain the embryonic transcriptional program and influence both somatic and germline development.

## MATERIALS AND METHODS

### Strains and maintenance

Nematode strain maintenance was as described previously (47). The wild-type strain N2 (Bristol) was used as the reference strain. The strains used are as follows: PFR506 *qaIs22[HA::wdr-5.1;Cbunc-119(+)];wdr-5.1(ok1417)III*, JA1597 *pdpy-30::cfp-1::GFP*, PFR572 *qaIs22[HA::wdr-5.1;Cbunc-119(+)];wdr-5.1(ok1417)III;pdpy-30::cfp-1::GFP*, PFR625 *qaIs22[HA::wdr-5.1;Cbunc-119(+)];wdr-5.1(ok1417)III;pdpy-30::cfp-1::GFP;sin-3(tm1276)I*, PFR510 *set-2(bn129)/qC1dpy-19(e1259)glp-1(q339)[qIs26]III*, PFR624 *cfp-1(tm6369) IV/nT1[unc?(n754),let-?](IV;V)*, PFR391 *wdr-5.1(ok1417)III* out-crossed twice, PFR590*sin-3(tm1276)* out-crossed twice, PFR630 *sin-3(tm1276)I/hT2[bli-4(e937)qIs48](I; III)*, PFR629 *set-2(bn129)III sin-3(tm1276)I*, PFR635 *set-2(bn129)III; cfp-1(tm6369)IV/nT1[unc?(n754),let?](IV;V)*, PFR636 *sin-3(tm1276)I;cfp-1(tm6369)IV/nT1[unc?(n754),let?](IV;V)*;dvIs70 [*hsp-16.2*p::GFP + *rol-6(su1006)]*, PFR593 *athp-1(tm4223)III*, obtained from the National Bioresource Project (Japan) and outcrossed twice, PFR696 *athp-1;cfp-1* obtained by crossing *cfp-1(tm6369)* males with *athp-1*(*tm4223)* hermaphrodites.

ChIP experiments used PFR253 *set-2(bn129)* (outcrossed 10X) and TM6369 *cfp-1(tm6369)* (outcrossed 4X). To generate the PFR624 balanced strain, *cfp-1(tm6369)* animals were crossed with wildtype males to generate heterozygote males that were crossed again with [unc] animals segregating from AV112 (*mre-11(ok179),V/nT1,[unc?(n754),let-?](IV;V))*. [unc] animals were selected and screened by PCR for the presence of the *cfp-1(tm6369)* deletion. To generate the PFR630 balanced strain, *sin-3(tm1276)* animals were crossed with wildtype males to generate *sin-3(tm1276)* heterozygote males that were crossed to GFP(+) animals segregating from*[F44E2.7(tm4302)/hT2]*. GFP(+) animals from this last cross were selected and screened by PCR for the presence of the *sin-3(tm1276)* deletion. Yeast two-hybrid strains were: EGY42 (MATa; *trp1, his3, ura3, leu2*); TB50 (MATα; *trp1, his3, ura3, leu2, rme1*). *cfp-1(tm6369)* is a deletion of 254 bp encompassing intron 4, exon 5 and intron 5 of *cfp-1*. It is predicted to produce a truncated CFP-1 protein of 374 aa lacking part of the conserved C-terminal domain. Primers used genotyping are listed in Supplementary Table S1.

### Immunoprecipitation for proteomics

Immunoprecipitations were performed on frozen embryos prepared by hypochlorite treatment from strains grown at 20°C on enriched NGM. For all immunoprecipitations, wildtype embryos (N2) were treated in parallel to serve as negative control in the mass spectrometry analysis. For HA ::WDR-5.1 immunoprecipitations, embryos from PFR506 were flash-frozen immediately after hypochlorite treatment. For CFP-1::GFP immunoprecipitations, PFR572 late stage embryos were obtained by incubating the embryos collected by hypochlorite treatment for 4 h prior to flash freezing in liquid nitrogen. For each condition embryos were ground to powder, resupended in IP buffer (50 mM HEPES/KOH pH 7,5; 300 mM KCl; 1 mM EDTA; 1 mM MgCl2; 0.2% Igepal-CA630 and 10% glycerol) containing complete protease inhibitors [Roche] and 1 mM PMSF, and sonicated. Protein extracts were recovered in supernantant following centrifugation at 20 000 g for 15 min at 4°C an 20°C and flash frozen in liquid nitrogen. Protein concentrations were estimated using the Bradford assay [Bio-Rad Protein Assay Dye]. For HA::WDR-5.1 immunoprecipitation, approximately 60 mg of total protein extract was incubated with protein G agarose beads [Sigma-Aldrich] in Bio-Spin Chromatography columns [Bio-Rad] for 30 min at 4°C on a rotator. Flow-through was collected and incubated with 240 μl slurry of anti-HA affinity matrix beads [Roche] in a fresh Bio-Spin Chromatography column for 90 min at 4°C on a rotator. The matrix was washed three times in IP buffer at 4°C and once in Benzo buffer (HEPES/KOH 50 mM pH 7,5; KCl 150 mM; EDTA 1 mM; MgCl_2_ 1 mM; Igepal-CA630 0.2%; glycerol 10%). The matrix was then incubated in 400 μl of Benzo buffer containing 2500 units of benzonase [Sigma] for 1 h at 4°C and washed three times in IP buffer. Four successive elutions were performed at 37°C for 15 min each with HA peptide (250 μg/ml in 240 μl of IP buffer). The first three eluates were pooled and concentrated 20 times (final volume 35 μl) using a 10 kDa Amicon Ultra centrifugal device [Merck]. 1/70 and 1/700 of this eluate were resolved on a 4–12% NuPage Novex gel [Thermo Fischer] and the gel either stained with SilverQuest staining kit [Thermo Fischer] or analyzed by western blot with anti-HA antibody [Covance HA.11, clone 16B12]. 33 μl of the eluate was diluted with 11 μl of LDS4X buffer [Thermo Fischer] and analyzed by mass spectrometry. For CFP-1::GFP immunoprecipitation, ∼70 mg of total protein were incubated in IP buffer with 100 μl of GFP-TRAP MA beads slurry [Chromotek] for 3 h at 4°C on a rotator. Beads were collected with a magnet, washed three times in IP buffer and one time in Benzo buffer, and then treated with benzonase. Eluates were recovered by incubation at 95°C for 10 min in 60 μl of LDS 1× buffer. 1/10 and 1/50 of this eluate were resolved on a 4–12% NuPage Novex gel [Thermo Fischer] and either stained with SilverQuest staining kit [Thermo Fischer] or analyzed by western blot with anti-GFP antibody [Sigma, 11814460001, clones 7.1 and 13.1] respectively, and 40 μl of the eluate was analyzed by mass spectrometry.

### Mass spectrometry-based proteomic analyses

Proteins were stacked in the top of a SDS-PAGE gel (4-12% NuPAGE, Life Technologies) and stained with Coomassie blue R-250 before in-gel digestion using modified trypsin (Promega, sequencing grade) as previously described (48). Resulting peptides were analyzed by online nanoLC-MS/MS (UltiMate 3000 and LTQ-Orbitrap Velos Pro, Thermo Scientific). For this, peptides were sampled on a 300 μm × 5 mm PepMap C18 precolumn and separated on a 75 μm × 250 mm C18 column (PepMap, Thermo Scientific). MS and MS/MS data were acquired using Xcalibur (Thermo Scientific). Peptides and proteins were identified using Mascot (version 2.5.1) through concomitant searches against Uniprot (*C. elegans* taxonomy), classical contaminants database (homemade) and the corresponding reversed databases. The Proline software (http://proline.profiproteomics.fr) was used to filter the results (conservation of rank 1 peptides, peptide identification FDR < 1% as calculated on peptide-spectrum match scores by employing the reverse database strategy, minimum peptide score of 25, and minimum of 1 specific peptide per identified protein group) before performing a compilation, grouping and comparison of the protein groups from the different samples. In WDR-5.1 mass spectrometry, 242 proteins were found with a spectral count (SC) WDR-5.1 ≥ 3 and a SC control (without WDR-5::HA transgene) = 0 or SC WDR-5.1/SC control ≥5 for at least one replicate; in CFP-1 mass spectrometry 178 proteins were found with a SC CFP-1 ≥3 and a SC control (without CFP-1::GFP transgene) = 0.

### Co-immunoprecipitation experiments

Co-immunoprecipitations with CFP-1::GFP were performed starting from 4 mg total protein embryonic extract from the strain containing the two transgenes CFP-1::GFP and HA::WDR-5.1. Samples were processed as in proteomic experiments. Co-immunoprecipitations with HA::WDR-5.1 were performed with the eluates sent to mass spectrometry analysis. Samples were processed as in proteomic experiments. Eluates were boiled in LDS sample buffer and analyzed on 4–12% NuPage Novex gels [Thermo Fischer] or Mini-PROTEAN TGX Stain-Free Precast gels [Bio-Rad] followed by western blotting. Antibodies used were: anti-GFP [Sigma, 11814460001, clones 7.1 and 13.1] (1/1000); anti-HA [Covance HA.11, clone 16B12] (1/2000); anti-HDA-1 [Novus Biologicals, 38660002] (1/2000); anti-DPY-30 [Novus Biologicals, 45110002] (1/5000); anti-ASH-2 (gift from B. Meyer) (1/4000); anti-MRG-1 antibody [Novus Biologicals, 35530002] (1/3000); anti-SIN-3 Q60131017(1/1000).

### Plasmids construction for Y2H

Plasmids used for expression of BD and AD fusions were derived from pEG202 (Clontech; Genbank Accession Number U89960) and pJG4–5 plasmids (Clontech; Genbank Accession Number U89961, respectively (49). Constructions were generated by cloning the cDNA of the gene of interest in the *Xho*I restriction site of the pEG202 and pJG4–5 plasmids using the Gibson method (50). CFP-1 truncations were obtained by the same reaction using the *cfp-1* cDNA sequence as template. PCR reactions were carried out using pHusion polymerase and primers listed in Supplementary Table S1. All products were verified by sequencing. pSH18–34, bearing a β-galactosidase gene under the control of four overlapping LexA operators was used as reporter vector (51).

### Interaction trap/two-hybrid system to identify interacting protein

Y2H assay is based on the LexA (BD)/B42 (AD) system (49). Cross-matings were performed in liquid phase (52). Competent haploid EGY42a cells were co-transformed with 1 μg of pSH18-34 (reporter vector) and 1 μg of BD construct. Competent TB50α cells were transformed with 1 μg of AD construct. Yeasts were selected for 3 days at 30°C on SD-UH (BD strains) and SD-W (AD strains) medium. Matings were performed overnight at 30°C in liquid YPAD (53). Cross-mating ensured that each hetero-interaction was tested twice (in both directions of the interaction matrix) and allowed the detection of homodimerisations. Diploids were amplified in selective liquid SD-UHW medium. For β-galactosidase assays, 50 μl of each diploids culture was inoculated (at OD_595 nm_ = 6) in 1 ml of pre-warmed (25°C) SGR-UHW medium supplemented with X-Gal (Thermo Scientific, #R0404) in Deepwell 96-well plates. Cultures were then incubated for 48 h at 25°C, centrifuged 5 min at 192g, resuspended in 300 μl, and transferred in flat bottom μClear Cellstar^®^ plates (Greiner Bio one) for scanning and phenotype assessment.

### Protein expression and GST pull-down assay

Full-length GST::CFP-1 and HIS_6_::SIN-3 fragments (1–738, 699–1507) were amplified using primers listed in Supplementary Table S1 and subcloned into pGEX-6-P1 [Sigma GE28-9546-48] and pPROEX HTa (gift from L. Terradot, MMSB, Lyon), respectively, using the Gibson method (50). All proteins were expressed in BL21 Rosetta 2 [Merck-Millipore 71402]. Bacteria were grown to OD_600_ 0.6 and protein expression induced with 1 mM IPTG at 16°C overnight. The pellet from 1 l of bacterial culture was resuspended in 10 ml lysis buffer (50 mM Tris pH 8,0 ; 300 mM NaCl; 0.1 mM EDTA; 0.1% Triton X-100 ; 0.05% NP-40; 1 mM MgCl_2_; 5% glycerol) containing protease inhibitor [Roche 05056489001]. Samples were sonicated on ice and centrifuged at 20 000 g for 20 min. For pulldown assays 200 ul of GST::CFP-1 supernatant was mixed with 800 ul HIS_6_::SIN-3[1–738] or HIS_6_::SIN-3[699-1507], respectively, and incubated overnight at 4°C on a rotating wheel. Samples were submitted to GST purification on a Biosprint 15 automat from Qiagen. Samples were washed three times with Lysis buffer and eluted with 50 ul of Lysis buffer containing 20 mM Glutathion. Eluted fractions were analyzed by western blot using a mouse anti-Histidine antibody [Sigma H1029] (1/3000) and Stain-Free gels (Bio-Rad).

### Brood size and embryonic lethality assays

For each strain, 10 L4 worms were isolated to single plates in the presence of excess food at 20°C, and allowed to develop into egg-laying adults overnight. Adult animals were then transferred to fresh plates every 12 h until they ceased laying eggs. Plates were scored for number of viable progeny and dead embryos that failed to hatch 24 h after removal of the mother.

### Fertility assay

Six independent lines were established from freshly thawed *sin-3(tm1276)* animals maintained as homozyotes, and homozygous *set-2(bn129)* and *cfp-1(tm6369)* animals obtained from balanced strains PFR 510 and PFR 624, respectively. For each line, six homozygous L4 stage animals were transferred to single plates with fresh *E. coli*, in the presence of excess food and cultivated at 25°C. From each generation, six worms were again picked to single plates until animals became sterile (fewer than 10 progeny/plate).

### Characterization of nuclear divisions in intestinal nuclei

Adult animals were treated with hypochlorite solution to obtained L1 synchronized larva. L1 larva were transferred to 25°C for 48 hrs, until they developed into adults. Young adults were stained with DAPI staining and analyzed with a Zeiss 710 Confocal Microscope. Experiments were performed in three independent replicates and intestinal nuclei from a total of 150 worms for each strain were scored.

### Comparison of gene expression changes in *cfp-1*, *set-2* and *sin-3* embryos

RNAs were extracted from wild-type, *cfp-1, set-2* and *sin-3* frozen early stage embryos prepared by hypochlorite treatment of young adults (>95% <200 cell stage). Two to three independent biological replicates were performed for each strain. RNAs were extracted with NucleoZol [Macherey-Nagel] according to manufacturer's instructions and treated with DNAse [Turbo-free DNAse, Ambion]. Integrity of RNA was assessed on Tape Station 4200 [Agilent]. RNA-seq librairies were generated at the GenomEast Platform [IGBMC, Strasbourg, France] using the directional mRNA-Seq SamplePrep [Illumina] and sequenced using the Illumina Hiseq 4000 technology. All RNA-seq data were mapped to the *C. elegans* reference genome (WS254) by RNA-STAR (Version 2.4.1d). Reads below a mapping score of 10 were filtered using SAMtools (Version 0.1.19). Of the 46 771 annotated genes, 20 183 were selected as protein coding genes and among them, 11 630 had sufficient read representation (baseMean > 10) for further analysis. The gene expression level in each sample was calculated by htseq-count (Version 0.7.2) and differential expression between the different strains was calculated with DESeq2 (54). Gene expression data are available at GEO with the accession GSE110072.

### Western blot analysis on histone marks

Embryos were obtained by hypochlorite treatment of adults grown on HB101 at 20°C and frozen in liquid nitrogen. Embryo pellets were resuspended in TNET buffer (50 mM Tris·HCl (pH 8), 300 mM NaCl, 1 mM EDTA, 0,5% Triton X-100 and protease inhibitors cocktail III [Merck]), lysed with zirconium beads [Lysing Matrix Y, MP Biomedicals #116960050] using a Precellys24 homogenizer and sonicated in a Bioruptor sonicator. Homogenates were centrifuged and supernatants aliquoted and frozen at −80°C. Total protein amount was quantified by the Bradford assay [Bio-Rad]. Serial dilutions of protein extracts were electrophoresed on 12% NuPage Novex gels for western blot analysis. Dilutions of wild type total protein extracts were analyzed to determine the upper limit of linearity of the following antibodies: anti-H3K4ac [Sigma, 07-539] (1/1000), anti-H3K9ac [Active Motif, 39137,39138] (1/2000), anti-H3K27ac [Active Motif, 39133] (1/2000), anti-H3K4me3 [Diagenode C15310003] (1/2000) and anti-H3 [Active Motif, 39163] (1/20 000). Each antibody was used on a separate blot loaded with the same extracts.

### Heat shock assay

Synchronized L4 staged worms carrying a *phsp-16.2::gfp* reporter (dvIs70) (55) were shifted at 33°C for 30 min in a water bath and allowed to recover at 20°C for 60 min. before observation. Experiments were repeated 5–8 times, with similar results. Fluorescent expression analysis were carried out on a Zeiss AxioPlan 2 equipped with Nomarski optics coupled to a camera (CoolSNAP, Roper Scientific). Non-heat shocked controls were equally overexposed.

### Chromatin immunoprecipitation

Wildtype, *cfp-1(tm6369)*, and *set-2(bn129)* mixed embryos were obtained by growing strains at 20°C in liquid culture using standard S-basal medium with HB101 bacteria. Strains were grown to the adult stage then bleached to obtain embryos, which were washed in M9, then frozen into ‘popcorn’ by dripping embryo slurry into liquid nitrogen. Chromatin immunoprecipitations and library preparations were conducted as in (56), using formaldehyde as a fixative for the H3K4me3 ChIPs (30 ug DNA, 2.5 ug antibody) and formaldehyde and EGS as fixatives for the SIN-3 (15 ug DNA, 2.5 ug antibody) and HDA-1 (30 ug DNA, 2.5 ug antibody) ChIPs. Approximately 10% *C. briggsae* chromatin extract was spiked into the *C. elegans* extract for the H3K4me3 ChIPs and 5% into the HDA-1 ChIPs. The HDA-1 antibody did not detect *C. briggsae* HDA-1 and so was not used for normalization. Two different antibodies to SIN-3 were raised through Strategic Diagnostics International by DNA immunization using aa427-576 (Q5986 and Q6013). Chromatin immunoprecipitations were conducted in duplicate with both SIN-3 antibodies in wild-type embryos; ChIP-seq patterns using these two SIN-3 antibodies were highly concordant (Supplementary Figure S1). Comparison of SIN-3 ChIP levels between wild-type and *cfp-1* mutant embryos were done using SIN-3 antibody Q5986. HDA-1 ChIPs were done using Novus 38660002/Q2354 and H3K4me3 ChIPs used Abcam ab8580. The age distributions of mixed embryo collections were in the following proportions (% <300 cell / % over 300 cell, average of the two replicates): H3K4me3 ChIPs: WT N2, 51/49; *cfp-1*, 59/51, *set-2*, 54/46. SIN-3 and HDA-1 ChIPs: WT N2, 48/52; *cfp-1*, 49/51. RNA-seq was performed on matched wild-type and *cfp-1* mutant embryo collections and sequencing libraries constructed as in (56). Libraries were sequenced using an Illumina HiSeq1500 and aligned to ce11 (WBCel235) *C. elegans* genome assembly with STAR aligner using Ensembl v90 gene annotation for splice aware alignment. Reads were counted using HTSeq method implemented in R and differential expression was assessed using DESeq2 method (54).

### SIN-3, HDA-1 and CFP-1::GFP ChIP-seq data processing

CFP-1::GFP (GEO GSE49870), SIN-3 and HDA-1 ChIP-seq reads were aligned to the ce11 assembly of the *C. elegans* genome using BWA v. 0.7.7 (57) with default settings (BWA-backtrack algorithm). The SAMtools v. 0.1.19 ‘view’ utility was used to convert the alignments to BAM format. Normalized ChIP-seq coverage tracks was generated using the BEADS algorithm (58). ChIP-seq peaks were called for SIN-3, HDA-1 and CFP-1::GFP in wild-type embryos using MACS2 v. 2.1.1 (59) with a *q*-value cut-off of 0.05 and fragment size of 150bp against summed ChIP-seq input (GEO GSE87524). Peaks overlapping non-mappable (GEM-mappability < 25%; (60) or blacklisted regions (https://gist.githubusercontent.com/Przemol/ef62ac7ed41d3a84ad6c478132417770/raw/56e98b99e6188c8fb3dfb806ff6f382fe91c27fb/CombinedBlacklists.bed) were discarded. Peak summits were extended 150 bp upstream and downstream, creating 300 bp peak regions. Intersecting regions from the two replicates were kept and extended to 300 bp to obtain confident peak calls. SIN-3 peak calls are the intersection of peaks obtained using the Q5986 and Q6013 antibodies. To determine factor overlaps, the 300 bp peak call regions from CFP-1, SIN-3 and HDA-1 were intersected, also keeping regions with only one factor. Regions from this ‘superset’ were rescaled to 300bp and each region annotated for overlap with a CFP-1, SIN-3, HDA-1 or MRG-1 peak (MRG-1 peaks were obtained from (61); Supplementary Table S2 gives these regions and their annotations. We used SeqPlots (62) for k-means clustering of CFP-1::GFP and H3K4me3 signals in wild-type, *cfp-1* mutants, and *set-2* mutants to separate CFP-1::GFP peaks into strong and weak COMPASS sites, and to visualise CFP-1::GFP, HDA-1 SIN-3, MRG-1 (GEO GSE50333) and H3K4me3 ChIP-seq tracks as heatmaps. The IGV Genome Browser (63) was applied to visualise example regions. Strong and weak COMPASS peaks were assigned to promoters and genes based on overlap with promoter annotations in (64); for genes with no mapped promoter, peaks were annotated as promoters and assigned to genes if they were within 500 bp of a Wormbase gene start. ChIP-seq data generated in this study is available at GSE114715.

### Spike-in normalization of H3K4me3 ChIP-seq

Sequencing reads from H3K4me3 ChIP and corresponding input samples were mapped to a concatenated reference genome sequence containing *C. elegans* ce11 and *C. briggsae* cb3 using BWA (62) and were then separated by species. Only reads that mapped uniquely (mapq> = 10) to non-blacklisted regions were kept. The spike-in ratios of *C. briggsae* to *C. elegans* chromatin present in the combined extract were calculated from the input sequence as *C. briggsae* read count divided by *C. elegans* read count. *C briggsae* H3K4me3 peaks were called from ChIP data using MACS2 (59) with default parameters. Scaling factors for each ChIP samples were calculated as corresponding spike-in ratio divided by *C. briggsae* H3K4me3 ChIP read count in peak regions in millions. These scaling factors were applied to *C. elegans* H3K4me3 ChIP raw coverage track. As a last step, ChIP background was removed from the scale coverage tracks by subtracting the mode and setting negative values to zero. The resulting tracks were used for visualization and analyzing H3K4me3 levels.

### ChIP-seq signal quantifications

To compare SIN-3 and HDA-1 binding between wildtype and *cfp-1* mutant embryos, we quantified average BEADS normalized, *z*-scored signal on different peak sets. The signal was obtained using the *bigWigSummary* utility from Kent library (65) implemented in *rtracklayer* package in R. These signals were represented as overlaid violin plots (showing signal distribution) and Tukey box plots (showing estimation of statistical significance of difference between medians as notches) (66). The comparison of H3K4me3 levels in wt, *cfp-1*, and *set-2* mutants was done in the same way, using spike-in normalized signal tracks for quantification.

## RESULTS

### Co-immunoprecipitation of subunits of the *C. elegans* SET1/COMPASS complex

**Figure 1.**
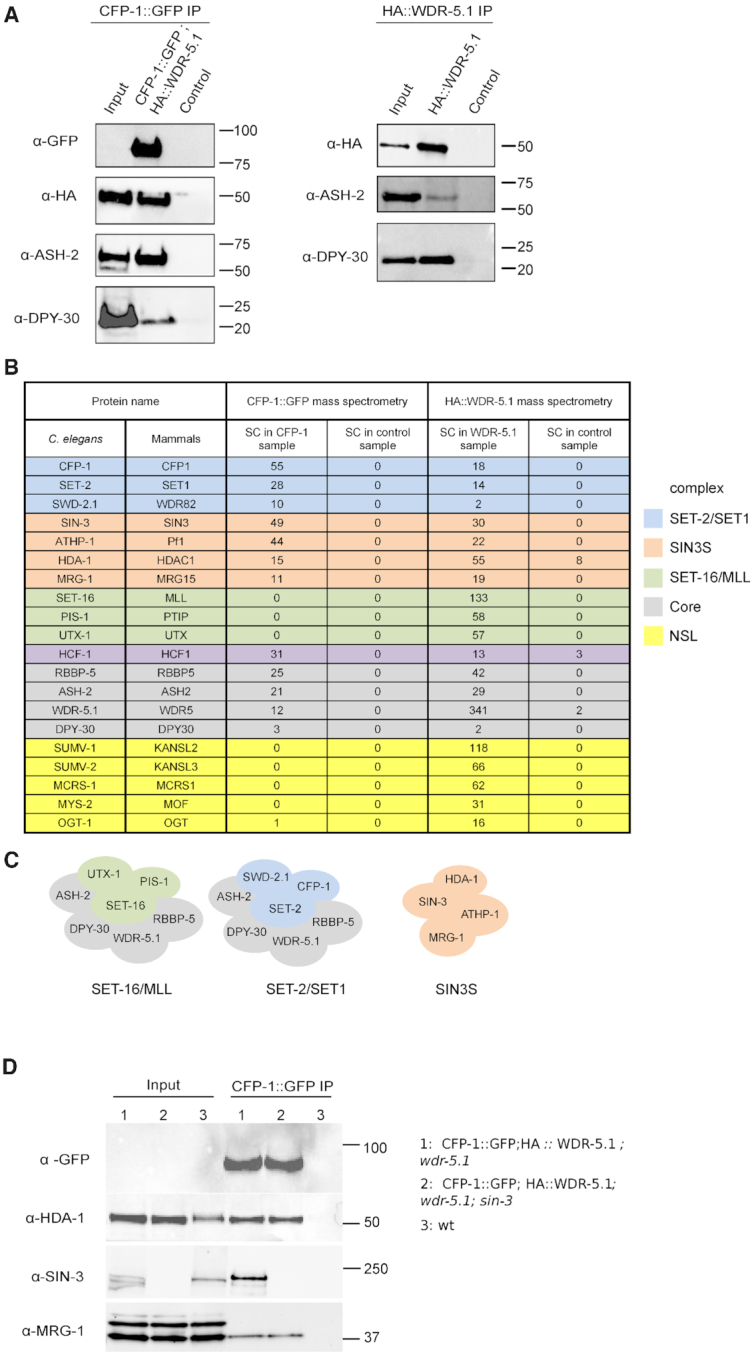
The conserved SIN3 complex copurifies with CFP-1 and WDR-5.1. (**A**) Co-IP of CFP-1, WDR-5.1, ASH-2 and DPY-30. Western blot analysis of CFP-1::GFP (left panel) and HA::WDR-5.1 (right panel) purified complexes from embryos. For CFP-1::GFP, immunoprecipitations were performed on 4 mg total protein extract, and loadings were 1/200 of total protein extract for input and 1/2 of total elution volume. For HA::WDR-5.1, samples used for mass spectrometry analysis were loaded as follows: 1/8000 of total protein extract for input and 1/250 of elution for α-ASH-2; 1/60 000 of total protein extract for input and 1/200 of elution for α-DPY-30. Control samples were prepared from a wild-type strain without either transgene. (**B**) List of selected proteins identified by mass spectrometry of CFP-1::GFP or HA::WDR-5.1 immunoprecipitations, and their mammalian homologue. Subunits specific to the SET-2/SET1, SET-16/MLL, SIN3S and NSL complexes are highlighted in blue, green, orange and yellow, respectively. SET1/MLL core complex subunits are highlighted in grey. HCF-1 copurifies with both SET-2/SET1 and SET-16/MLL complexes. SC; Spectral Counts. (**C**) Cartoon representation of SET-2/SET1, SET-16/MLL and SIN3S complexes; subunits are highlighted as in (B). (**D**) Co-IP of CFP-1, HDA-1, SIN-3 and MRG-1. CFP-1::GFP immunoprecipitations were carried out on 5 mg total protein and analyzed by western blot using anti-HDA-1, SIN-3 and MRG-1 antibodies. Loadings were 1/5000 of total protein extract for input and 1/5 of total elution volume for anti-GFP and anti-HDA-1, 1/1000 of total protein extract for input and 1/5 of total elution volume for anti-SIN-3 and anti-MRG-1.

We used a proteomics approach to characterize *C. elegans* COMPASS-like complexes and search for associated proteins. In addition to the catalytic subunit, SET1/COMPASS complexes contain the core components ASH2, RbBP5, WDR5 and DPY30, and the unique subunits CFP1/CXXC and WDR82 (3,22,33,67–69). Using strains containing two previously described transgenes, CFP-1::GFP and HA::WDR-5.1 (38,70) (Supplementary Figure S2), we found that CFP-1::GFP coprecipitated HA::WDR-5.1, and that HA::WDR-5.1 coprecipitated CFP-1::GFP (Figure 1A, Supplementary Figure S3A). In addition, probing individual precipitates with anti-ASH-2 and anti-DPY-30 antibodies revealed the presence of both proteins (Figure 1). Because single or low copy tagged SET-2 protein could not be detected, we were unable to confirm its presence in the IP experiments.

The above experiments show that in embryos, CFP-1::GFP and HA::WDR-5.1 tagged proteins associated with each other *in vivo* and co-immunoprecipitate native ASH-2 and DPY-30, consistent with their incorporation into a SET1-related complex. To define SET1/MLL complexes and identify additional associated proteins, we undertook mass spectrometry-based proteomic characterization of CFP-1::GFP and HA::WDR-5.1 immunoprecipitates. We reasoned that WDR-5.1, a core component of SET1/MLL and other chromatin complexes, should immunoprecipitate both SET1 and MLL-related complexes (21,71,72), while CFP-1 should specifically immunoprecipitate SET1, but not MLL-related complexes (2,3). Both tagged proteins were detected as unique bands in immunoprecipitates obtained using either anti-GFP or anti-HA antibodies (Supplementary Figure S3B), and as predominant bands by silver-staining (Supplementary Figure S3C). Tandem mass spectrometry (MS/MS) based proteomic analyses of the immunoprecipitates and comparison with eluates from negative controls identified both common and unique binding partners of CFP-1 and WDR-5 (Figure 1B, see Supplementary Table S3 for a full list). We found that CFP-1::GFP and HA::WDR-5.1 immunoprecipitates contained all common subunits of SET1/MLL complexes, including Swd1/RBBP-5, Bre2/ASH-2, and Sdc1/DPY-30. The orthologue of human host cell factor HCF1, a transcriptional regulator associated with COMPASS-like and other chromatin-associated complexes (73) was also identified in both immunoprecipitates. WDR-5.1 additionally coprecipitated specific components of an MLL-related complex including the MLL-like histone methyltransferases SET-16, the histone H3K27 demethylase UTX-1 and PIS-1 (74). Conversely, CFP-1 specifically immunoprecipitated SET-2/SET1 and SWD-2.1/WDR82, but not SET-16/MLL, consistent with it being a unique component of SET1, but not MLL complexes (10,69,75). These results define distinct SET-2/SET1 and SET-16/MLL complexes in *C. elegans* embryos (Figure 1C).

WDR-5.1 immunoprecipitates also contained subunits of the NSL histone acetyltransferase (HAT) complex, consistent with findings in other organisms (44,76–79). We identified the MOF homologue MYS-2, OGT-1, MCRS-1 and the NSL2 and NSL3 homologues SUMV-1 and SUMV-2, respectively. Homologues of two other NSL components found in other organisms, MBD-R2/PHF20 and NSL1/KANSL1, are not found in the *C. elegans* genome (80). Therefore, as in other species, *C. elegans* WDR-5.1 is found in the NSL complex as well as COMPASS/MLL complexes.

### *C. elegans* homologs of the Sin3S complex copurify with CFP-1 and WDR-5.1

Four additional proteins, SIN-3, HDA-1, MRG-1 and ATHP-1, were reproducibly identified as top hits in both HA::WDR-5.1 and CFP-1::GFP immunoprecipitates (Figure 1B and Supplementary Table S3). These are homologs of subunits of the Rpd3/Sin3 small complex in yeast (Rpd3/Sin3S) and SHMP in mammalian cells (Figure 1B, see below) (81,82). In yeast and other organisms a second type of Rpd3/Sin3 complex is found (Rpd3L in yeast) defined by the presence of SAP30, SDS3, and other subunits (83–87). *C. elegans* does not harbor a SAP30 subunit, but has a single counterpart of SDS3, SUDS-3, which was absent from our proteomics analysis with CFP-1 and WDR-5. We will therefore refer to the complex of SIN-3, HDA-1, MRG-1 and ATHP-1 as the *C. elegans* SIN3S complex (Figure 1C).

Functions ascribed to Rpd3/Sin3 complexes are varied and appear to be context dependent. Although typically referred to as corepressor complexes due to the presence of a histone deacetylase subunit, Rpd3/Sin3 complexes have been associated with both activation and repression of gene expression. In addition, the yeast Rpd3/Sin3S complex has been shown to repress cryptic transcription initiation in transcribed regions and to suppress antisense transcription initiation at promoters (81,88).

Sin3 proteins, which lack known DNA-binding motifs or enzymatic activity, are characterized by the presence of four paired amphipathic helices (PAH) with structural similarity to Myc family transcription factors (89), and a conserved HDAC-interacting domain (HID) (90). While mammals contain two Sin3 proteins (Sin3A and Sin3B) that share both overlapping and distinct functions (91–94), SIN-3 is the only *C. elegans* homologue. It contains a HID domain, and a single PAH most closely related to the highly conserved PAH1 in mammals (95) (Supplementary Figure S3D). *C. elegans* HDA-1 is one of three class I histone deacetylases (HDACs) in *C. elegans* and a component of several other chromatin complexes, as in other species (96,97). MRG-1, the *C. elegans* counterpart of the chromo-domain (CD) protein Eaf3/MRG15, is also found in additional chromatin complexes (98–103), and ATHP-1 (AT Hook plus PHD finger transcription factor), a counterpart of Rco1/Pf1, contains two AT Hooks and a Forkhead-associated (FHA) domain that are not found in either Rco1 or Pf1 (Supplementary Figure S3D).

Western blot analysis on CFP-1::GFP immunoprecipitates using antibodies against endogenous MRG-1, HDA-1, and SIN-3 proteins confirmed the interactions between CFP-1 and SIN3 complex components detected by mass-spectrometry (Figure 1D and Supplementary Figure S3E). Interaction between CFP-1 and SIN-3 was also observed in young adults, showing that is not specific to embryos (Supplementary Figure S3F). We also confirmed that HDA-1 co-precipitates with WDR-5.1 (Supplementary Figure S3G). We further found that interaction of HDA-1 and MRG-1 with CFP-1 is not dependent on endogenous SIN-3, as both proteins are found in CFP-1 immunoprecipitates obtained from *sin-3* mutant extracts (Figure 1D). We conclude that CFP-1 physically interacts with a Sin3S complex, but may also interact with HDA-1 and MRG-1 in other contexts.

### Subunits of the SET-2/SET1 and SIN3S complex physically interact

**Figure 2.**
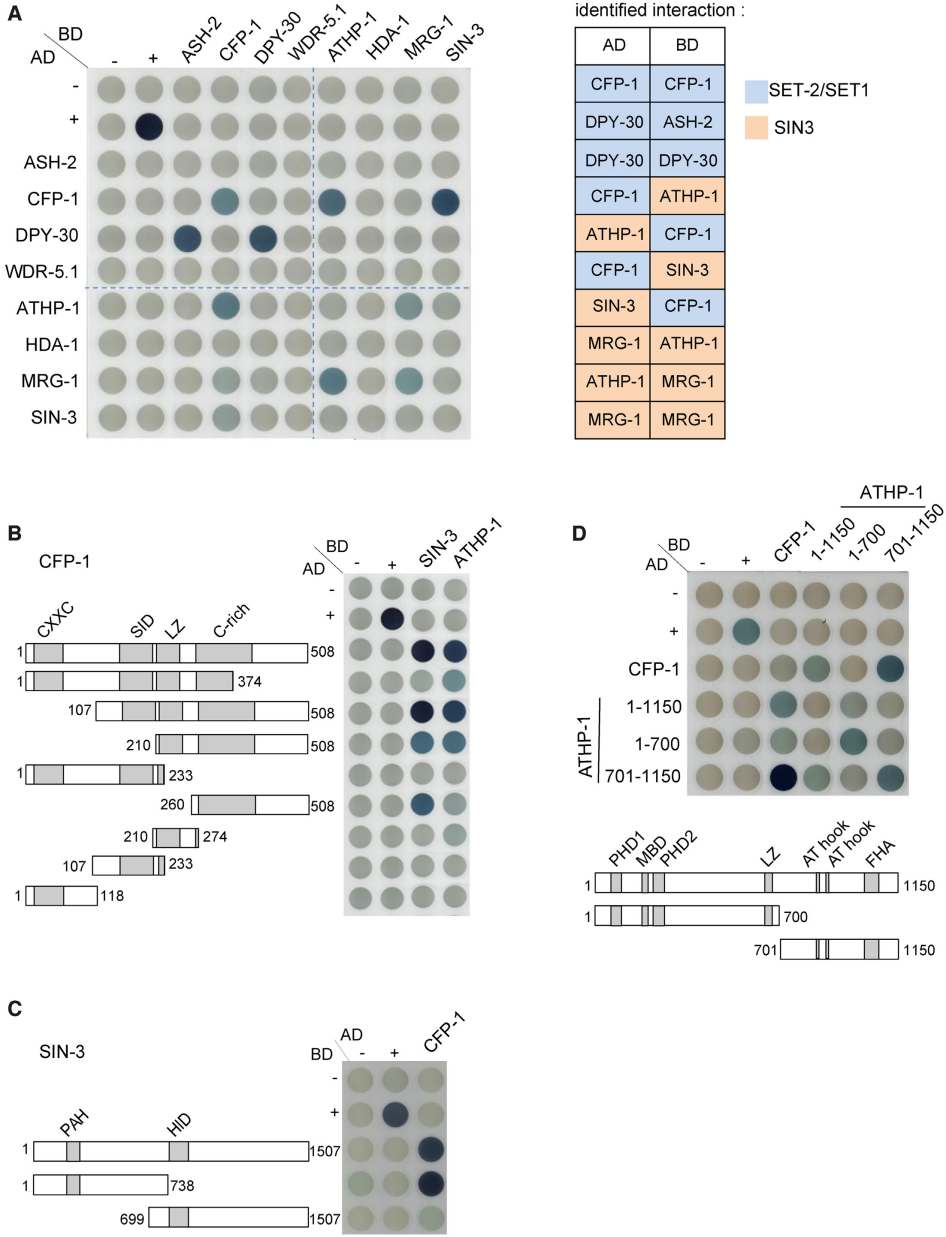
Interaction mapping between subunits of the SET-2/SET1 and SIN3S complexes by Y2H. (**A**) An interaction matrix was obtained by cross-mating of yeast haploid strains expressing different subunits as AD or BD fusion proteins. Positive control (+) is barnase/barstar interaction (133). Matings that lead to visually detectable staining in two independent experiments are reported in the tabular form on the right. Failure to detect the DB DPY-30/AD ASH-2 interaction is most likely due to DPY-30 homodimerization in the context of the DB domain fusion interfering with ASH-2 binding. (**B**) Interaction matrix of full length and truncated CFP-1 tested against full length SIN-3 and ATHP-1. CFP-1 truncations were constructed as BD and AD fusions, as indicated. (C, D) Interaction matrix of full length CFP-1 tested against N- and C-terminal fragments of SIN-3 (**C**) and ATHP-1 (**D**).

We used a yeast two-hybrid assay to assess potential physical interactions between components of the SIN3S and SET-2/SET1 complexes (49). A full-length cDNA of each SET-2/SET1 and SIN3S complex subunit was cloned into vectors to express DNA-binding (DB) and activation domain (AD) fusions. Western blot analysis confirmed expression of all cDNAs with the exception of *set-2* (Supplementary Figure S4A). Testing pairwise interactions of BD and AD fusions by cross-mating, we detected interaction between DPY-30 and ASH-2, and DPY-30 homodimerization within the SET-2/SET1 complex, consistent with studies in other systems (20,45) (Figure 2A). In addition, we detected CFP-1 homodimerisation (Figure 2A). Within the SIN3S complex, we observed an interaction between MRG-1 and ATHP-1, and MRG-1 homodimerization (Figure 2A). Importantly, we found that CFP-1 interacted with the SIN3S complex components ATHP-1 and SIN-3.

### The C-terminal domain of CFP-1 is necessary and sufficient for interaction with SIN-3

Mammalian CFP1 contains an N-terminal PHD domain that recognizes methylated H3K4, a Zn finger CXXC domain that binds to unmethylated CpG dinucleotides, a Set1 interaction domain (SID), a coiled-coiled leucine zipper (LZ) domain, and a cysteine-rich C-terminal domain (1,4,104–106) (Supplementary Figures S3D and S5). *C. elegans* CFP-1 contains all of these except for the PHD domain. To identify the domains that mediate interaction of CFP-1 with SIN-3 and ATHP-1, we expressed different regions of CFP-1 and tested their ability to interact with full length SIN-3 and ATHP-1 by Y2H as described above. Western blot analysis confirmed the expression of all CFP-1 constructs with the exception of DB 1–374 (Supplementary Figure S4B). We found that neither the N-terminal CXXC domain, nor the SID domain, were required for interaction with either SIN-3 or ATHP-1 (Figure 2B). The cysteine-rich C-terminal domain fragment interacted with SIN-3, and a larger fragment additionally containing the LZ domain was sufficient for interaction with ATHP-1. These results indicate that CFP-1 binds to SIN-3 through a region containing the cysteine-rich domain, and that interaction with ATHP-1 requires both this region and the LZ domain.

Using Y2H, we further showed that CFP-1 interacts with SIN-3 through its N-terminal region that contains the conserved PAH domain (Figure 2C), and with ATHP-1 through its C-terminal region (Figure 2D). Pull-down assays confirmed a direct interaction between the N-terminal fragment of SIN-3 and CFP-1 (Supplementary Figure S3H). Altogether, these results support the finding that CFP-1 physically interacts with the SIN3S complex.

### Phenotypic similarity of SIN3S and SET-2/SET1 complex mutants

**Figure 3.**
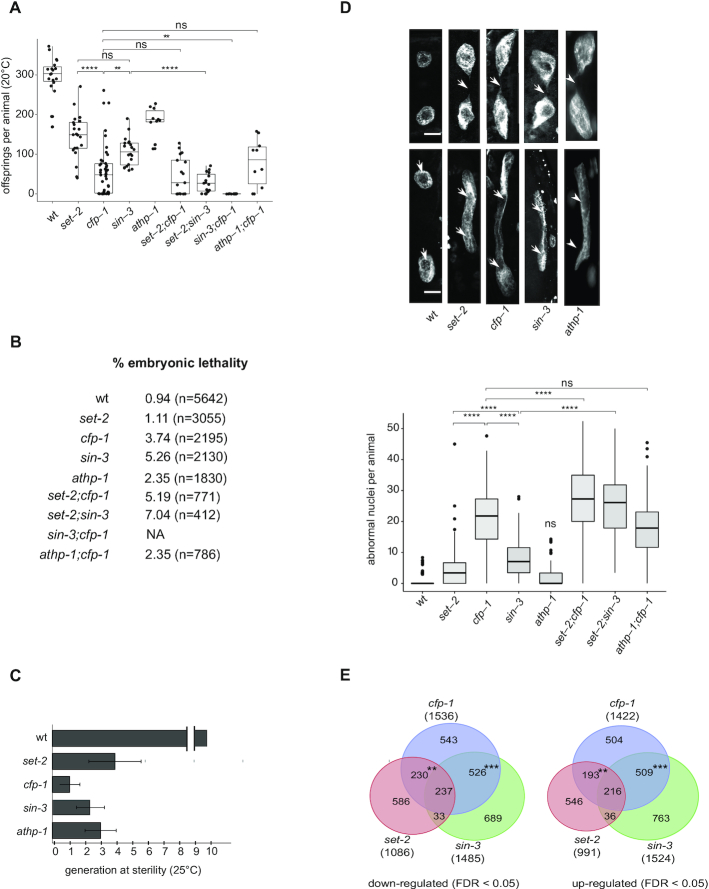
Loss of function of SET1/COMPASS and SIN3S complex components results in similar phenotypes and affects steady state gene expression. (**A**) Total number of progeny of single and double mutant animals of the given genotype. Multiple comparison was done using Wilcoxon (with Bonferroni correction) post hoc method following a significant Kruskal Wallis test; asterisks indicate a significant difference: *P < 0.05, **P < 0.01, ***P < 0.001, ****P < 0.0001. (**B**) Embryonic lethality of single and double mutant strains; n = total number of embryos scored. (**C**) Fertility assays of *set-2, cfp-1, sin-3* and *athp-1* mutants grown at 25°C. Scoring was based on three to four biological replicates, with 6 independent lines each. Wildtype animals can be maintained for more that 40 generations without loss of fertility. (**D**) Confocal images of DAPI-stained intestinal nuclei from young adults. Examples of nuclear division abnormalities giving rise to thin chromatin bridges (arrows, top panel), or thick chromatin dense regions connecting two nuclei (arrowheads, bottom panel). Box plots show the total number of segregation defects (thin and thick chromatin bridges) per animal in single and double mutants of the given genotype (*n* = 150 worms for each strain). Multiple comparison was done using Wilcoxon (with Bonferroni correction) post hoc method following a significant Kruskal Wallis test as in (A). (**E**) Venn diagram showing the overlap between *cfp-1*, *set-2* and *sin-3* downregulated and upregulated genes. *P*-value for overlap between commonly misregulated genes in pairwise comparisons was calculated using Fisher's exact test in R: **P-value < 10^−10^ and ***P-value < 10^−100^.

The physical interactions between CFP-1 and SIN3S complex components suggest that they may function in shared processes. To investigate this, we compared phenotypes of *set-2*, *cfp-1*, *sin-3* and *athp-1* mutants alone or in double mutant combinations, using null or strong loss of function alleles for all four genes (34,41,107). Similar to *set-2* mutants, we observed that *cfp-1, sin-3* and *athp-1* mutants also have reduced brood size at 20°C (32,34,41). *cfp-1* mutants showed extreme variability, with some animals showing a near-wild-type brood size, and others being completely sterile (Figure 3A). The brood size of *set-2; cfp-1* and *athp-1; cfp-1* double mutants is not reduced further compared to *cfp-1* single mutants, suggesting that SET-2 and ATHP-1 do not have CFP-1 independent fertility functions. However, *sin-3* fertility functions are independent or partially redundant with *set-2* and *cfp-1*, as brood size of *set-2; sin-3* is lower than that of the single mutants, and *cfp-1; sin-3* double mutants showed a fully penetrant sterility that precluded scoring additional phenotypes (Figure 3A and data not shown). All single and double mutants also have a low level of embryonic lethality (Figure 3B).

*set-2* mutants show transgenerational sterility at the stressful temperature of 25°C (32,38,41), and we found that *cfp-1, sin-3* and *athp-1* mutants also show this phenotype at 25°C. As expected, *set-2* mutants became sterile at generation F3–F4 (Figure 3C). We observed that *sin-3* and *athp-1* mutants become sterile at the F2–F3 generation, whereas the progeny of *cfp-1* mutants that were shifted to 25°C at the L4 stage were sterile (F1 generation).

We also observed that *cfp-1*, *set-2*, *sin-3* mutants have chromosome segregation defects in intestinal cells that become binucleate in the L1 stage (108). A similar trend was observed in *athp-1* mutants. Intestinal nuclei in adult animals were frequently connected by either thin or thick chromatin bridges in single mutants, and often completely failed to separate in *cfp-1* single, and *set-2;cfp-1*, *set-2;sin-3* and *athp-1;cfp-1* double mutants (Figure 3D). In summary, the similar phenotypes and genetic interactions suggest that SET-2/SET1 and SIN3 complexes are, at least in part, functionally linked in the germline and soma.

### Similar steady-state gene expression changes in *set-2, cfp-1* and *sin-3* mutants

To ask whether SET-2/SET1 and SIN3 complexes have common roles in gene expression, we next performed RNA-sequencing (RNA-seq) on staged *cfp-1*, *set-2* and *sin-3* mutant embryos. Using DESeq2 (FDR < 0.05), we derived lists of differentially expressed genes in each mutant background, finding a similar number that were up- or down-regulated (Supplementary Table S4, Supplementary Figure S6). Consistent with the phenotypic similarities, we observed that gene expression changes detected in all three mutants show significant overlap (Figure 3E). Additionally, pairwise comparisons revealed gene expression changes shared only by *cfp-1* and *sin-3* mutants or only by *cfp-1* and *set-2* mutants, whereas *set-2* and *sin-3* do not show a specific association (Figure 3E). These shared patterns suggest that CFP-1 can act independently with COMPASS and SIN3S (Figure 3E).

Gene ontology (GO) term analysis showed enrichment for biological pathways related to translation, reproduction and development in all three mutant contexts (Supplementary Table 5). Downregulation of genes related to reproduction most likely reflects maternally inherited transcripts whose expression is altered in the germline of these mutants (41).

### CFP-1 and SET-2/SET1 are needed for H3K4me3 at promoters

**Figure 4.**
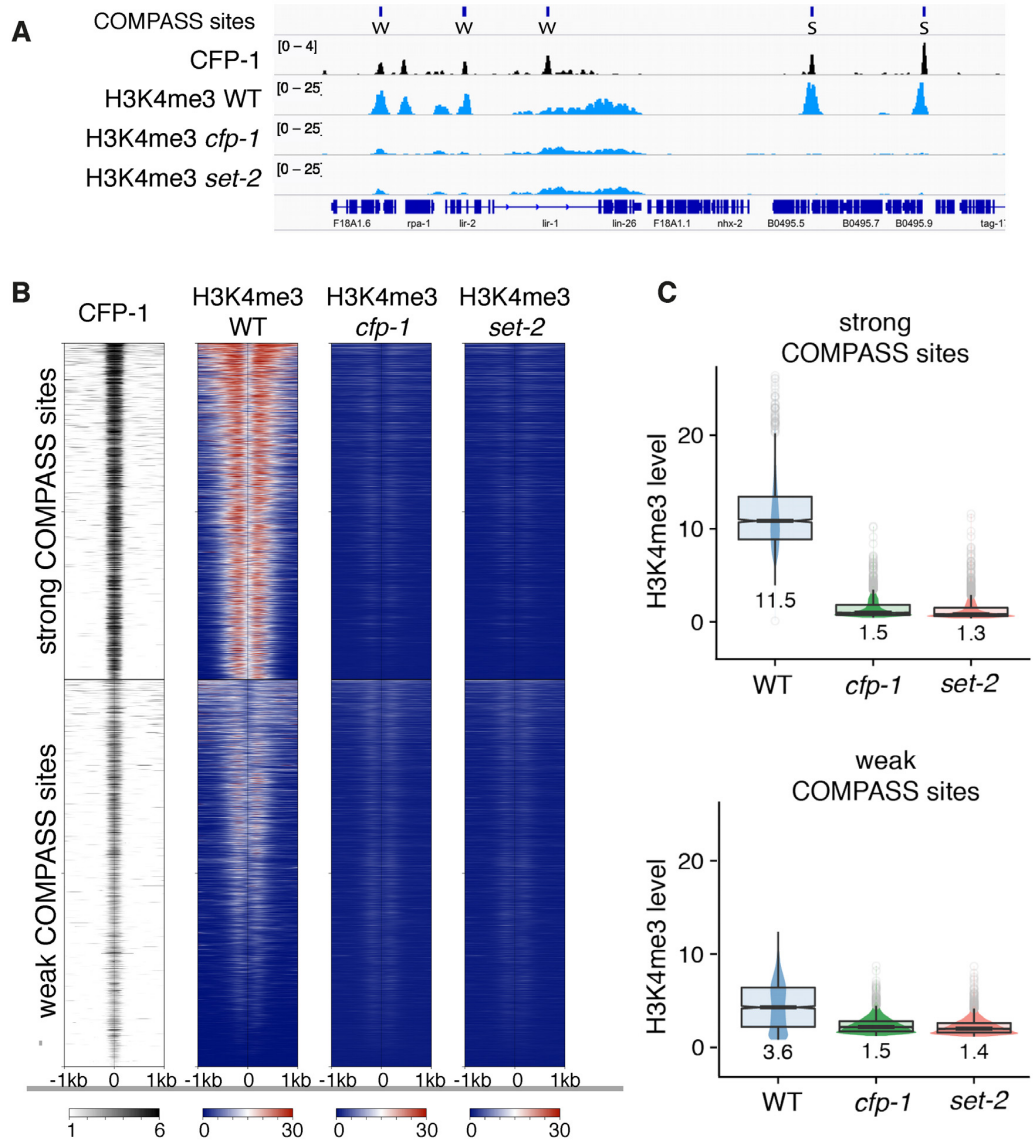
H3K4me3 at strong and weak COMPASS targets is dependent on CFP-1 and SET-2. (**A**) IGV browser view showing z-score BEADS normalized ChIP-seq signals of CFP-1::GFP, and H3K4me3 in wildtype, *cfp-1*, and *set-2* mutant embryos normalized to *C. briggsae* spike-in (see Methods). Top track shows locations of strong (S) and weak (W) COMPASS targets. (**B**) Heatmap of CFP-1::GFP, and H3K4me3 in wildtype, *cfp-1*, and *set-2* mutant embryos, using same tracks as in (A). (**C**) Quantification of H3K4me3 signal (*C. briggsae* normalized) in strong and weak COMPASS targets.

Previous studies showed that *cfp-1* or *set-2* inactivation results in greatly reduced global levels of H3K4me3 (32,34) (Supplementary Figure S7A). In addition, CFP-1 binding sites were shown to map to H3K4me3 marked promoters (70). To determine the roles of the two proteins on the pattern of H3K4me3 at CFP-1 sites, we compared H3K4me3 ChIP-seq signals in wildtype with those in *cfp-1* and *set-2* null mutant embryos, using a spike-in control for normalization. We observed strong reduction of H3K4me3 at CFP-1 sites in both *cfp-1* and *set-2* mutants (Figure 4A and Supplementary Figure S7B, see Methods). Using hierarchical clustering, we observed two classes of CFP-1 binding sites in wild-type embryos, both of which have reduced H3K4me3 in the two mutants. Sites with a high level of CFP-1 are strongly marked by H3K4me3, whereas sites with lower CFP-1 levels have low H3K4me3 marking (Figure 4A, B). We define the high H3K4me3 level sites as strong COMPASS targets, and the low level CFP-1 sites as weak COMPASS targets. The finding that the genomic distribution of H3K4me3 is similarly reduced in *cfp-1* and *set-2* mutants confirms that CFP-1 is needed for SET-2 activity at promoters.

Similar to findings in ES cells (1), we observed no clear relationship between gene expression changes in *cfp-1* mutants and promoter association of CFP-1. Both up- and down-regulated genes showed weak enrichment for CFP-1 binding, and only 2% of genes with CFP-1 peaks had significantly altered expression (Supplementary Figure S8A). The lack of a strong association between binding and gene expression change is consistent with evidence from various systems suggesting that additional factors, such as stress and age, influence the impact on transcription (11,109,110). For example, in yeast, SET1 acts primarily as a repressor of stress-induced transcription, with little effect of its loss under steady state conditions (9,11). In line with this, we found that *hsp-16.2::*GFP induction following heat shock was consistently stronger and more widespread in *cfp-1*, *sin-3* and *set-2* adult animals compared to wildtype, supporting a possible role in moderating the level of induced gene expression (Supplementary Figure S8B).

### SIN3 complex components colocalize with CFP-1 at promoter regions

**Figure 5.**
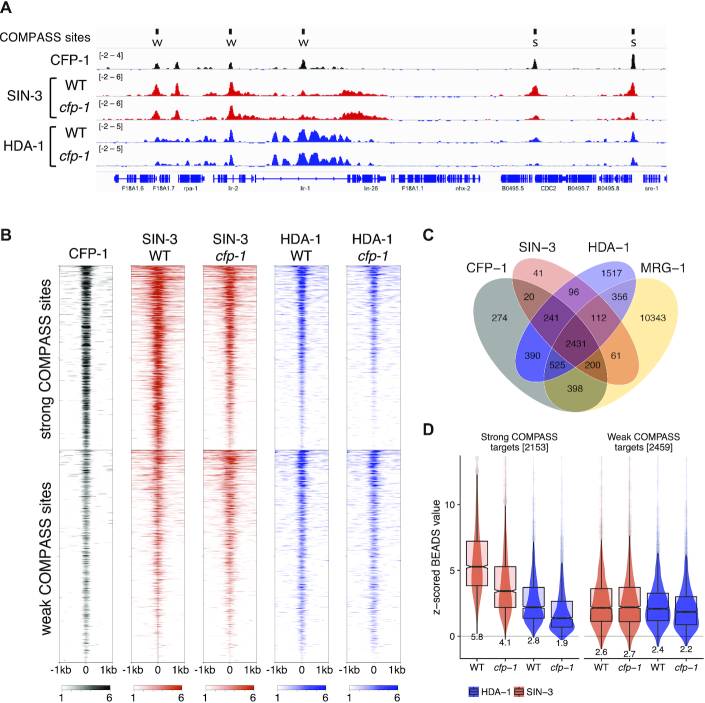
SIN-3 and HDA-1 require CFP-1 for recruitment to strong COMPASS targets. (**A**) IGV browser view showing *z*-scored BEADS normalized ChIP-seq signals from mixed embryos of indicated strains. Top track shows locations of strong (S) and weak (W) COMPASS targets. (**B**) Heatmap of *z*-scored BEADS normalized ChIP-seq signals from mixed embryos over strong and weak COMPASS targets in indicated strains. (**C**) Venn diagram showing overlap of CFP-1, SIN-3, HDA-1 and MRG-1 ChIP-seq peaks. (**D**) Quantification of normalized z-scored SIN-3 and HDA-1 signal in wildtype and *cfp-1* mutants at strong and weak COMPASS targets.

We next investigated how the distribution of SIN3 complex components relates to that of CFP-1. Using ChIP-seq analysis of SIN-3 in wild-type embryos, we observed that the pattern of SIN-3 binding was highly similar to that of CFP-1; 90% of SIN-3 peaks overlap a CFP-1 peak and 77% of these sites are found at promoters (Figures 5A-C, Supplementary Figure S9). In addition, as observed for CFP-1, SIN-3 levels are higher at strong COMPASS targets than at weak COMPASS targets (Figure 5B). We next determined the distribution of the SIN3 complex component HDA-1. We observed that HDA-1 was also present at most CFP-1 binding sites, with similar levels at strong and weak COMPASS targets (Figure 5A, B and Supplementary Figure S10). HDA-1 is additionally found at many sites that lack CFP-1 and SIN-3, presumably through its association with other proteins and complexes (Figure 5C) (97). Using previously published ChIP-seq data mapping MRG-1 in embryos (61), we observed weak enrichment at promoters and a broad distribution on the gene bodies of many actively transcribed genes (Supplementary Figure S9). In addition, 59% of sites harboring peaks of SIN-3, HDA-1 and CFP-1 (*n* = 2707) overlap an MRG-1 peak (Figure 5C). We also observed that SIN3 complex components SIN-3 and HDA-1 have a broader distribution than CFP-1 and are weakly enriched on gene bodies (Supplementary Figure S9). The finding that CFP-1 and SIN3 complex components extensively colocalize at promoter regions supports connected functions.

### CFP-1 facilitates SIN-3 binding to H3K4me3 enriched promoter regions

The similarity in binding patterns together with our biochemical studies showing that CFP-1 physically associates with the SIN3S complex suggests a potential role in SIN3 chromatin recruitment. To investigate this possibility, we used ChIP-seq to map SIN-3 and HDA-1 binding in *cfp-1* mutant embryos. We observed that strong COMPASS targets had significantly reduced levels of both SIN-3 and HDA-1 in *cfp-1* mutants compared to wildtype (Figures 5A, B, D). In contrast weak COMPASS targets were largely unaffected (Figures 5A, B, D). HDA-1 sites that lack CFP-1 or SIN-3 binding and random genomic regions also showed no change in SIN-3 or HDA-1 levels in *cfp-1* mutants (Supplementary Figure S11). Together with the physical interaction results, we conclude that CFP-1 promotes recruitment of the SIN3 complex to strong COMPASS target sites.

## DISCUSSION

In this study we identify a physical and functional interaction between CFP-1, the chromatin targeting subunit of the highly conserved SET1/COMPASS complex, and a SIN3S histone deacetylase complex similar to yeast Rpd3S and mouse SHMP containing SIN-3, HDA-1, MRG-1 and ATHP-1. We show that CFP-1 mediates interaction with the SIN3S complex through a direct interaction with SIN-3 and that it promotes recruitment of SIN-3 and HDA-1 at promoters. The interactions with SIN3S and other chromatin regulators identified by proteomics indicate that CFP-1 function extends beyond targeting the COMPASS complex to chromatin and support a role for CFP-1 in coordinating the activities of distinct chromatin complexes.

Our biochemical data also provide evidence for distinct SET-2/SET1 and SET-16/MLL related complexes in *C. elegans*, consistent with the presence of multiple H3K4 HMT complexes in metazoans (19). WDR-5.1 and CFP-1 both immunoprecipitated the core complex proteins RBBP-5, ASH-2, and DPY-30, as well as the SET1/COMPASS subunits SET-2/SET1 and SWD-2.1/WDR82 (111–113). However, WDR-5.1, but not CFP-1, immunoprecipitated unique subunits of the previously identified SET-16/MLL complex including the histone H3K27 demethylase UTX-1, and PIS-1 (74). WDR-5.1 also co-immunoprecipitated the NSL complex, consistent with its role as a central hub in several additional chromatin-associated complexes. Interestingly, in mammalian cells NSL has been shown to promote H3K4me2 activity by MLL1 (44), and we identified the single MLL1 homologue SET-16 with NSL subunits in our experiments, suggesting this activity may be conserved in *C. elegans*.

Y2H analyses showed that CFP-1 interacts with both SIN-3 and ATHP-1 subunits of the SIN-3 complex. We observed a direct interaction between CFP-1 and SIN-3 that is dependent on the C-terminus of CFP-1 containing the conserved cysteine-rich domain, and the N-terminal domain of SIN-3 containing the highly conserved PAH domain. The PAH1 and PAH2 domains of mammalian SIN3 have been shown to facilitate SIN3 recruitment by transcription factors (95), however little is known about the function of the cysteine-rich C-terminal domain of mammalian CFP1. Although the C-terminal region of ATHP-1 that interacts with CFP-1 is not found in either yeast or mammalian proteins, it contains an FHA domain shown to mediate interaction between other transcription factors and the SIN3 complex in mammalian cells (114), raising the possibility that other proteins may fulfill a similar function in other species. Y2H analyses also confirmed physical interactions within SIN3 and SET-2/SET1 complexes and showed CFP-1 homodimerization, supporting studies suggesting dimerization of CFP1 within the SET1A/B complexes in human cells (28).

Supporting a functional link between COMPASS and SIN3S complexes, we observed similar germline and somatic phenotypes in *set-2, cfp-1*, *sin-3* and *athp-1* mutant animals, as well as shared gene expression changes between *set-2, cfp-1* and *sin-3*. We also found that CFP-1 regulates gene expression independently with SET-2 and SIN-3. These findings, together with the physical association and promoter co-occupancy of CFP-1 and SIN-3, suggest that CFP-1 may modulate gene expression by independently recruiting SET-2/SET1 and SIN3S complexes to promoters. Interestingly, the yeast CFP1 homologue Spp1 acts at meiotic recombination sites independently of *Set1*, and is found in at least two distinct Set1-independent complexes (115–117). COMPASS independent functions of Spp1 are also supported by studies showing that dissociation of Spp1 from SET1/COMPASS repurposed its function to promote transcriptional memory (118).

Previous genetic studies further support both unique and common functions for components of these complexes and other proteins isolated in our proteomics approach. For example, inactivation of the SIN3 complex subunits *sin-3* and *mrg-1*, the NSL complex subunits *sumv-1* and *sumv-2*, and the SET-2/SET1 complex subunits *cfp-1, wdr-5.1* and *dpy-30*, but not *set-2*, can all suppress the synthetic multivulval (SynMuv) phenotype resulting from mutations in repressive chromatin factors (119,120). A subset of these genes, including *sin-3*, *mrg-1, wdr-5.1* and *dpy-30*, but neither *set-2* nor *cfp-1*, also suppress the larval lethality resulting from inactivation of *lin-35*/Rb in a sensitized background (121). We found that CFP-1 promotes binding of SIN-3 and HDA-1 at strong COMPASS dependent promoters, consistent with direct recruitment dependent on the activity of the SET-2/SET1 complex. SIN-3 binding at weak COMPASS regions was not affected. Interestingly, in mammalian cells COMPASS and MLL complex subunits have been shown to cofractionate with SIN3, further supporting a connection between the complexes (122,123).

A prevailing view is that the regulatory functions of both SET1/COMPASS and Rpd3/Sin3 complexes are context dependent, but these functions are not well understood (10,75). For example, knock-out of Sin3 in different systems results in both gene activation and repression (124–127), and we observed no clear relationship between gene expression changes and SIN-3 binding. Similarly, loss of CFP1 or SET1 in a wide range of different systems causes surprisingly few gene expression changes relative to the number of genes marked by H3K4me3, with no clear relationship between expression and marking under steady state or induced conditions (1,10,11,109,128,129). We also observed no reproducible change in bulk acetylation in *sin-3*, *cfp-1* or *set-2* mutants (Supplementary Figure S7A). SIN-3 and CFP-1 could alter gene expression through transient changes in acetylation and methylation dynamics that cannot be detected in the context of dividing embryos (130). Alternatively, or in addition, properties such as local nucleosome density and dynamics could also be affected (131). Our finding that expression from a heat-shock inducible promoter increased in the absence of CFP-1, SIN-3 or SET-2 is consistent with proposed roles in response to external stimuli in yeast (11). Interestingly, recent data suggests that AMPK signaling may regulate SET-2/SET1 complex activity in response to stress (132). Future work on defined loci will be needed to understand these regulatory functions. Because of the high degree of conservation between mammalian and *C. elegans* SET1/COMPASS and Rpd3/Sin3 complexes, our finding that they functionally interact through a direct physical interaction with CFP-1 contributes towards understanding the complexity of interactions between chromatin associated proteins with distinct activities.

## DATA AVAILABILITY

Data generated in this study are available from GEO: gene expression data, GSE110072; ChIP-seq data, GSE114715. Other ChIP-seq datasets used are CFP-1::GFP (GSE49870; Ref. (70) and MRG-1 (GSE50333; Ref. from (61)

## Supplementary Material

gkz880_Supplemental_Files

## Notes

Present address: Ron A.-j Chen, School of Molecular and Cellular Biology, University of Leeds, Leeds, UK.
