# Supplementary material for: Physical and functional interaction between SET1/COMPASS complex component CFP-1 and a Sin3S HDAC complex in C. elegans
Source: Nucleic Acids Res. 2019 Oct 11;47(21):11164–80. doi: 10.1093/nar/gkz880 (PMC6868398; doi:10.1093/nar/gkz880)
Supplement: gkz880_Supplemental_Files [file gkz880_supplemental_files.zip › 19_08_Supplemental_Information.pdf]

Supplemental Information

Supplementary Figure 1. Concordance of ChIP-seq signals obtained using two different anti-SIN-3 antibodies. Shown are genome-wide correlations of average signal in 1kb bins.

Supplementary Figure 2 (related to Figure 1). Expression of HA*::wdr-5.1* and *cfp-1*::GFP and rescue of the *wdr-5.1(ok1417)* mutation by the HA::*wdr-5.1* construct. (A) anti-HA immunostaining of early and late-stage embryos expressing HA*::wdr-5.1*, and live imaging of *cfp-1*::GFP expressing embryos. (B) Western blots analysis on mixed-stage embryos from wildtype, HA*::wdr-5.1; wdr-5.1(ok1417)* and *wdr-5.1(ok1417)* animals. HA::*wdr-5.1* rescues the loss of H3K4me3 caused by *wdr-5.1* deletion. Membranes were incubated with anti-H3K4me3, anti-H3 and anti-actin antibodies, as indicated.

Supplementary Figure 3 (related to Figure 1). Immunoprecipitation of CFP-1::GFP and HA::WDR-5.1 and validation of interactions. (A) HA::WDR-5.1 copurifies CFP-1::GFP. HA::WDR-5.1 immunoprecipitation was carried out on 8 mg total protein from the strain containing the two transgenes HA::WDR-5.1 and CFP-1::GFP and analyzed by western blot using anti-HA or anti-GFP antibodies. Control samples were prepared from a wild-type strain without either transgene. (B) Western blot analysis of CFP-1::GFP and HA::WDR-5.1 immunoprecipitations used for mass spectrometry analysis. Immunoprecipitation using anti-GFP antibodies was performed on 70 mg of total protein extract. Quantities loaded were 1/700 of total protein extract (100 μg) as input, and 1/50 of total elution. Immunoprecipitation using anti-HA antibodies was performed on 60 mg of total protein extract and pooled elutions were concentrated. 1/4000 of total protein extract (15 μg) was loaded as input or unbound fraction, and 1/700 of concentrated and non-concentrated elutions were loaded. (C) Silver staining of CFP-1::GFP (left) or HA::WDR-5.1(right) immunoprecipitated samples from *C. elegans*

embryonic extracts. (*) indicates band corresponding to CFP-1::GFP and HA::WDR-5.1. (D) Domain conservation of *C. elegans* and mammalian SIN3, MRG-1/MRG15 and ATHP1/Pf1 proteins. (E) Specificity of SIN-3 antibodies. Western blots and Stain-Free gels (Bio-Rad) of total embryonic extract using two distinct anti-SIN-3 antibodies, (Q5986 and Q6013) in wildtype and *sin-3(tm1276)* mutants. Both antibodies detect a SIN-3 specific band (*) at the expected MW that is absent from *sin-3* mutants. (F) Co-IP of CFP-1 and SIN-3 from young adults. CFP-1::GFP immunoprecipitations were carried out using anti-GFP antibodies and blots revealed with anti-GFP and anti-SIN-3 antibodies. Arrow points to the band corresponding to SIN-3. (G) Western blot analysis of HA::WDR-5.1 immunoprecipitation used for mass spectrometry analysis revealed using anti-HDA-1 or anti-HA antibodies. Loadings were 1/60 000 of total protein extract (1 µg) for input and 1/200 of concentrated elutions. (H) GST pull-down assay between GST::CFP-1 protein and HIS_6::SIN-3 N-terminal domain (aa 1-738; left) or HIS_6::SIN-3 C-terminal domain (aa 699-1507; right). Western blot was revealed with anti-Histidine antibodies. Arrow points to HIS_6 fusion protein.

Supplementary Figure 4 (related to Figure 2). Expression of BD and AD fusion proteins in yeast. Total protein extracts of BD (bait) and AD (prey) haploid yeast strains were analyzed by western blot and revealed using anti-HA antibodies.

Supplementary Figure 5 (related to Figure 2). Sequence alignments of *H. sapiens* and *C. elegans* CFP1 and Rpd3/Sin3S complex subunits. (A) Alignement of CFP1. Black boxes show conserved PHD, CXXC, SID, LZ and cysteine-rich (C-rich) C-terminal domains. (B) alignement of MRG15 and MRG-1 chromo domains (CD). (C) Alignment of Pf1 and ATHP-1 PHD domains. (D) Alignment of Sin3B and SIN-3. Black boxes show conserved PAH1 and HID domains. Dashed lines at aa positions 699 and 738 show position of truncations used in

GST pull-down experiments in Fig. 2D. All alignments were carried out with Clustal Omega Multiple Sequence Alignment software.

Supplementary Figure 6 (related to Figure 3). *cfp-1*, *set-2* and *sin-3* misregulated genes. Volcano plots show log2 of fold change in expression between mutant and wildtype (wt) embryos (~95% 200-cell stage or less) plotted against statistical significance (log10 adjusted p value (padj) for all genes with a baseMean > 10 (black dots). Horizontal lines mark a significance cut-off of padj = 0.05.

Supplementary Figure 7 (related to Figure 4). H3K4me3 and H3 acetylation levels in different mutant backgrounds. (A) Western blot analysis of total protein extracts for wt, *sin-3(tm1276), cfp-1(tm6369)* and *set-2(bn129)* embryos. Membranes were incubated with indicated antibodies. Each antibody was used on a separate blot loaded with the same extracts.
Blot is representative of three or more experiments carried out with distinct biological replicates. (B) Comparison between library and input normalized to *C. briggsae* spike-in normalized H3K4me3 signals. The y-axes of wildtype and *cfp-1* mutant are matched in the two comparisons and the the same genomic region is shown for the two normalization methods. Standard normalization to input and library size (top) artifically inflates weak signals.

Supplementary Figure 8 (related to Figure 5). Correlation between steady state gene expression changes and promoter association of CFP-1, and increased induced expression expression of a reporter in live mutant animals. (A) Venn diagrams showing overlap between genes up- and down-regulated in mixed stage *cfp-1* mutant embryo and CFP-1 promoter peaks. Differentially expressed (DE) genes were defined as those having an adjusted p-value (FDR) of <0.05 and base mean >10. Peaks were assigned to genes based on overlap with coding promoter

annotation from (141). P-values shows significance of overlap (based on Fisher's test) and *e* denote expected number of overlaps based on hypergeometric distribution. (B) Increased induction of *hsp-16* in *cfp-1*, *set-2* and *sin-3* mutant animals. Adult animals were heat-shocked 33°C 30 min and mounted for observation under a light fluorescence microscope 90 min after heat-shock. Images show GFP expression before (top panel) and after (bottom panel) heat shock. Images of control non-heat shocked animals are overexposed. Similar results were obtained in 5 or more independent experiments with 50 or more animals for each condition.

Supplementary Figure 9 (related to Figure 5). Comparison of CFP-1, SIN-3, HDA-1, and MRG-1 binding patterns. (A) IGV browser view of showing z-scored BEADS normalized ChIP-seq signals from wild-type embryos (B) Heatmap of z-scored BEADS normalized ChIP-seq signals from mixed embryos over strong and weak COMPASS targets in wild-type embryos. (C) Gene profile plots of CFP-1, SIN-3, HDA-1, and MRG-1 signal anchored at strong COMPASS promoters (top) and weak COMPASS promoters (bottom). SIN-3, HDA-1, and MRG-1 signals are broader than those of CFP-1.

Supplementary Figure 10 (related to Figures 4 and 5). Comparison of SIN-3 and HDA-1 signals on different sets of regions in wildtype and *cfp-1* mutant embryos. Average signal in indicated regions was quantified from normalized z-scored SIN-3 and HDA-1 ChIP-seq tracks in wildtype and *cfp-1* mutants. HDA-1 unique regions are HDA-1 peaks that do not overlap with a CFP-1 or SIN-3 peak. Random regions are random 300bp genomic regions that do not overlap with a CFP-1, SIN-3, or HDA-1 peak. Random regulatory sites are random chromatin accessibility sites (300bp centered on the site) reported in (141) that do not overlap with a CFP-1, SIN-3, or HDA-1 peak.

Supplementary Figure 11 (related to Figure 5). CFP-1, SIN-3, and HDA-1 co-associate at promoters. Protein coding promoters (n=13,596) from (141) were separated in to three clusters: strong COMPASS sites, weak COMPASS sites, non-COMPASS sites based on overlap with CFP-1 peaks. Indicated ChIP-seq signal was quantified (top) and plotted (bottom) on the three clusters.

Supplementary Table 1. List of primers.

Supplementary Table 2. Peaks and annotations. All regions containing a peak for CFP-1::GFP, SIN-3, or HDA-1, annotated for overlap with each of these factors, MRG-1 (from (105); GEO GSE50333), annotated promoters (141) and Wormbase gene starts.

Supplementary Table 3 (related to Figure 1). Lists of top 300 proteins identified by mass spectrometry of CFP-1::GFP or HA::WDR-5.1 immunoprecipitations. Pep: number of identified peptides; SC: Spectral Counts; SSC: Specific Spectral Counts; WSC: Weighted Spectral Counts.

Supplementary Table 4 (related to Figure 3). List of misregulated genes (FDR<0.05)

Supplementary Table 5 (related to Figure 3). GO term analysis of up- or down regulated genes in *cfp-1*, *set-2* and *sin-3* mutants. Analysis was performed using the NCBI webtool DAVID.
