# Supplementary material for: Physical and functional interaction between SET1/COMPASS complex component CFP-1 and a Sin3S HDAC complex in C. elegans
Source: Nucleic Acids Res. 2019 Oct 11;47(21):11164–80. doi: 10.1093/nar/gkz880 (PMC6868398; doi:10.1093/nar/gkz880)
Supplement: gkz880_Supplemental_Files [file gkz880_supplemental_files.zip › Suppl_Figures_merged.pdf]

Fig. S1

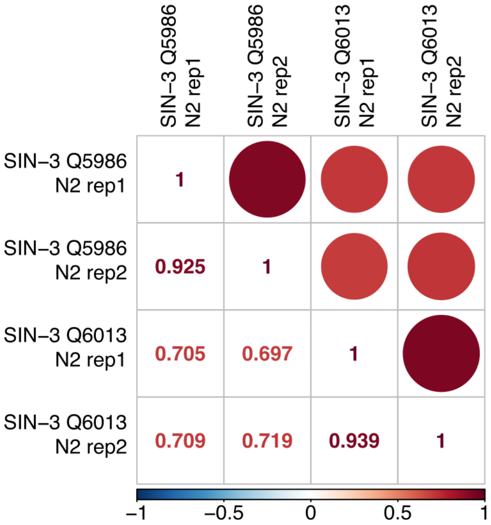

SIN−3 Q5986 N2 rep1
SIN−3 Q5986 N2 rep2
SIN−3 Q6013 N2 rep1
SIN−3 Q6013 N2 rep2
1
0.925
0.705
0.697
0.709
0.719
0.939
−1
−0.5
0
0.5

Fig. S2

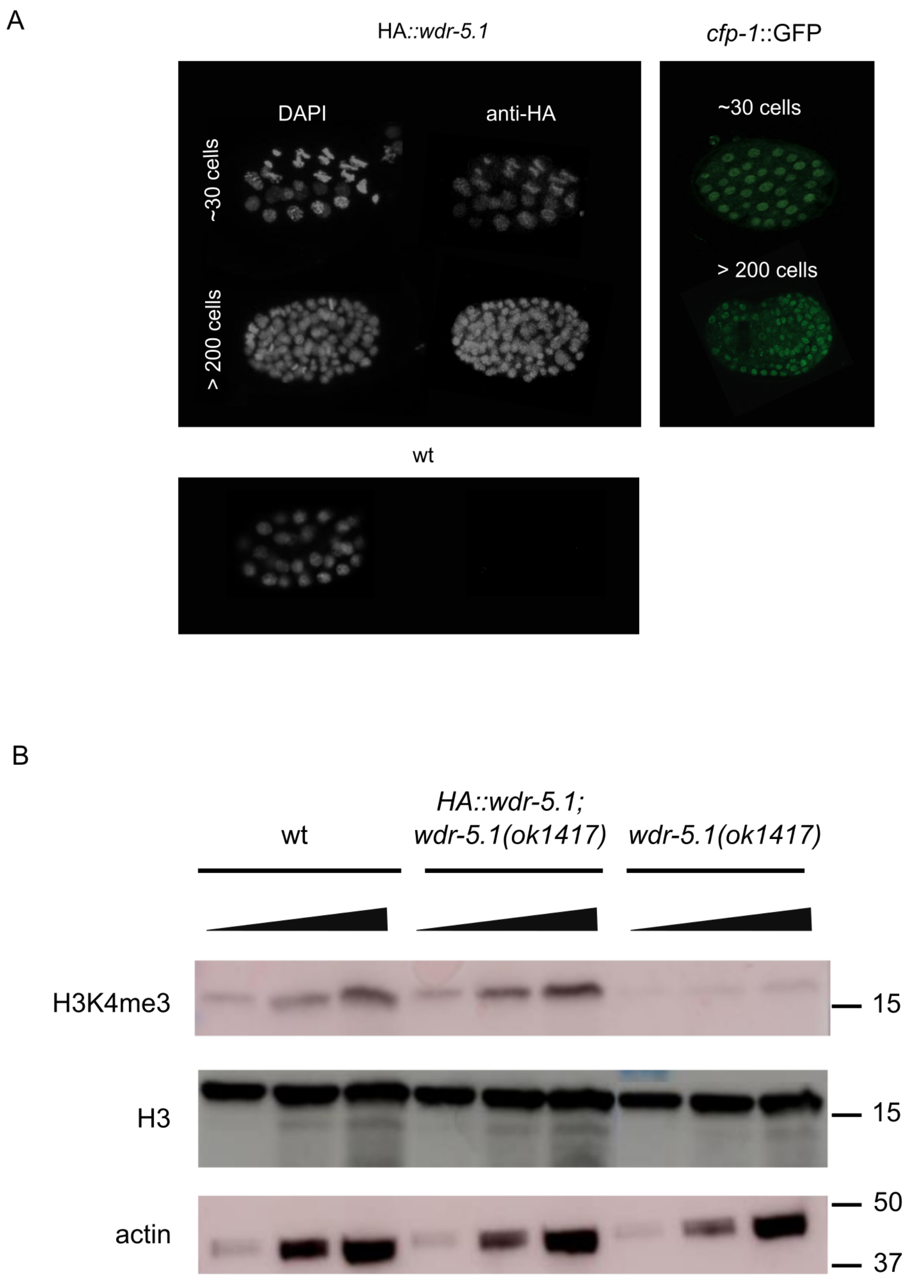
A
HA::wdr-5.1
cfp-1::GFP
DAPI
anti-HA
~30 cells
> 200 cells
~30 cells
> 200 cells
wt
B
wt
HA::wdr-5.1;
wdr-5.1(ok1417)
wdr-5.1(ok1417)
H3K4me3
15
H3
15
actin
50
37

H

Fig. S4

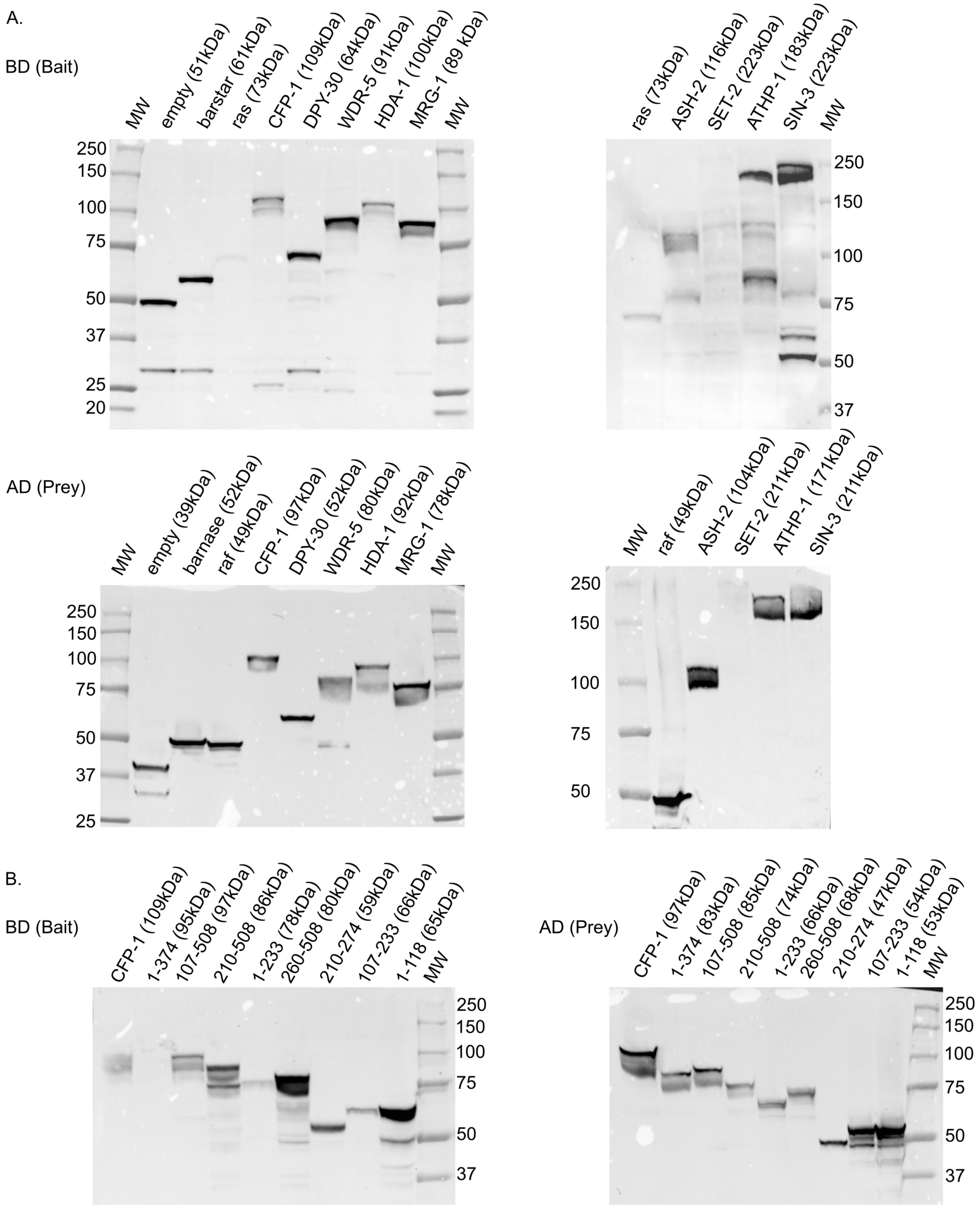

(--kDa) : theoritical size

Fig. S5

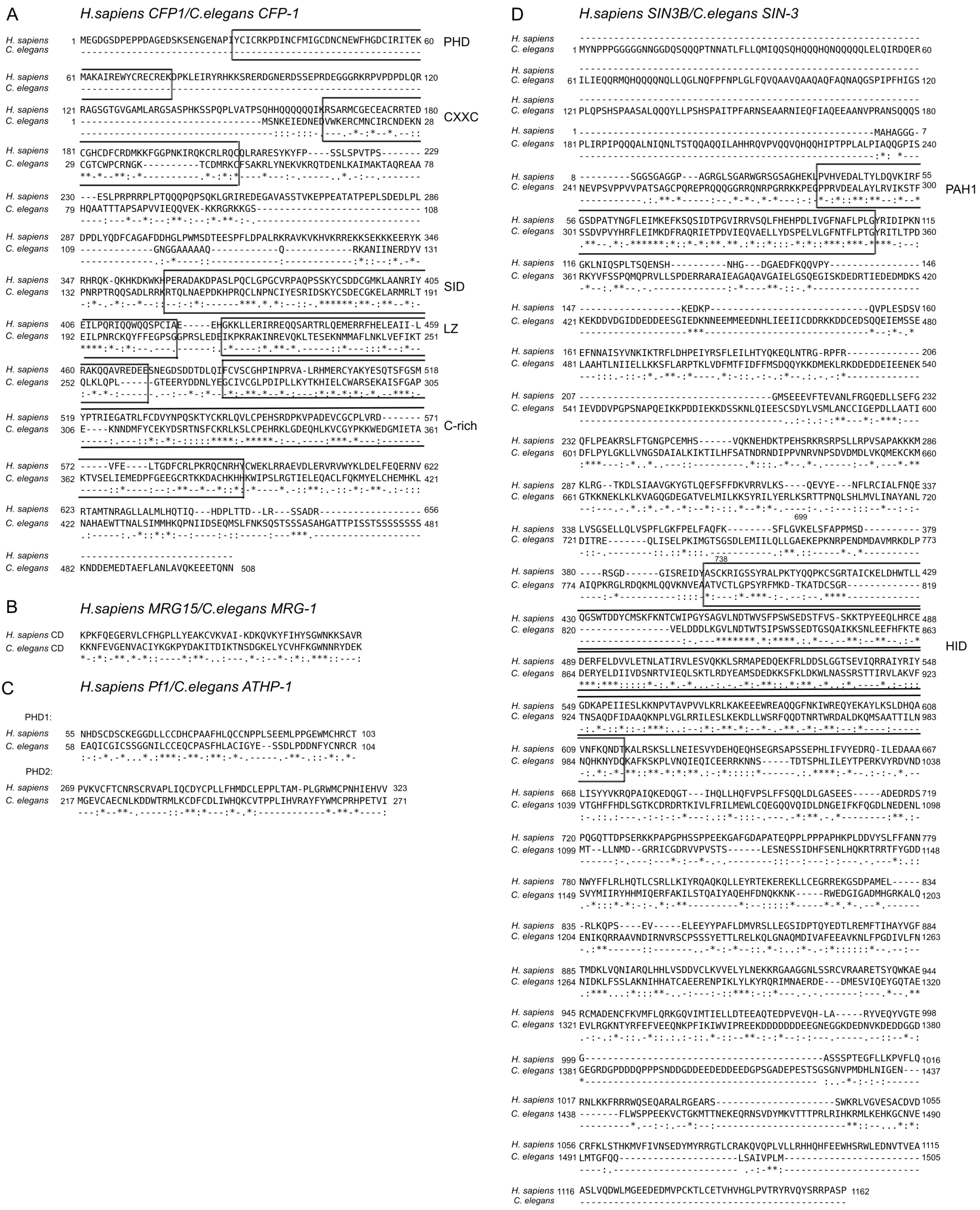
A
H.sapiens CFP1/C.elegans CFP-1
PHD
CXXC
SID
LZ
C-rich
B
H.sapiens MRG15/C.elegans MRG-1
C
H.sapiens Pf1/C.elegans ATHP-1
PHD1:
PHD2:
D
H.sapiens SIN3B/C.elegans SIN-3
PAH1
HID

Fig. S6

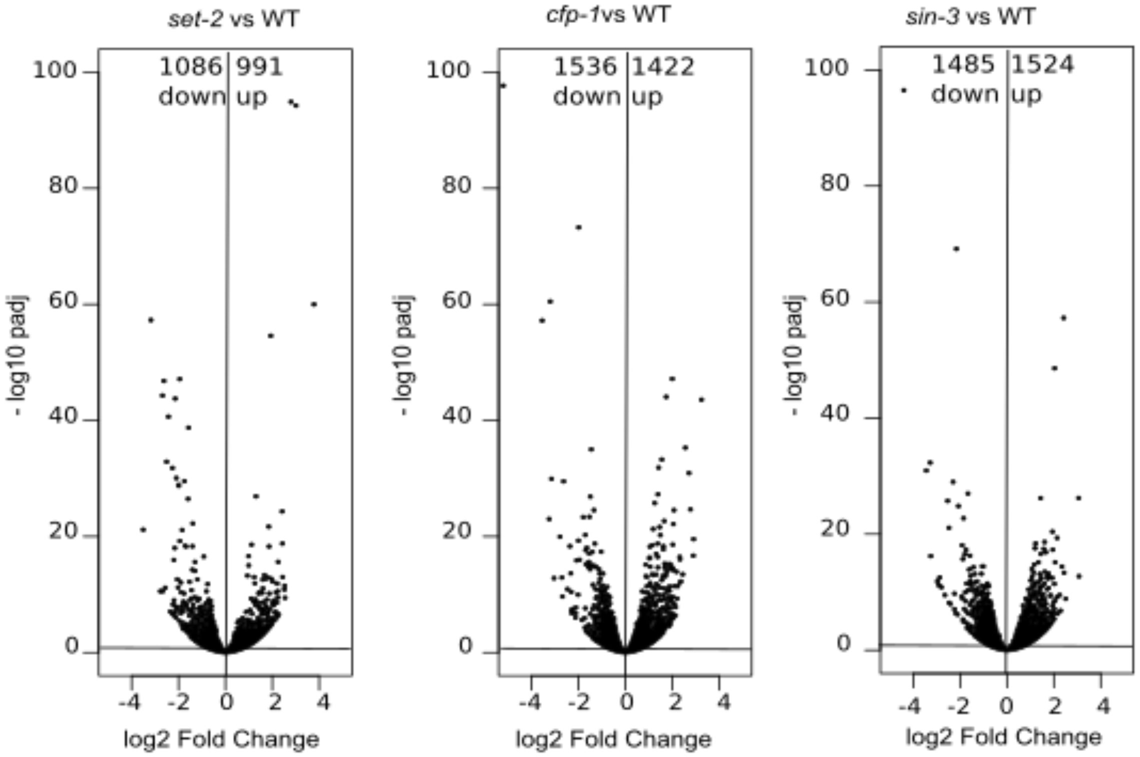

set-2 vs WT
1086 991
down up
cfp-1vs WT
1536 1422
down up
sin-3 vs WT
1485 1524
down up
- log10 padj
log2 Fold Change

Fig. S7

A

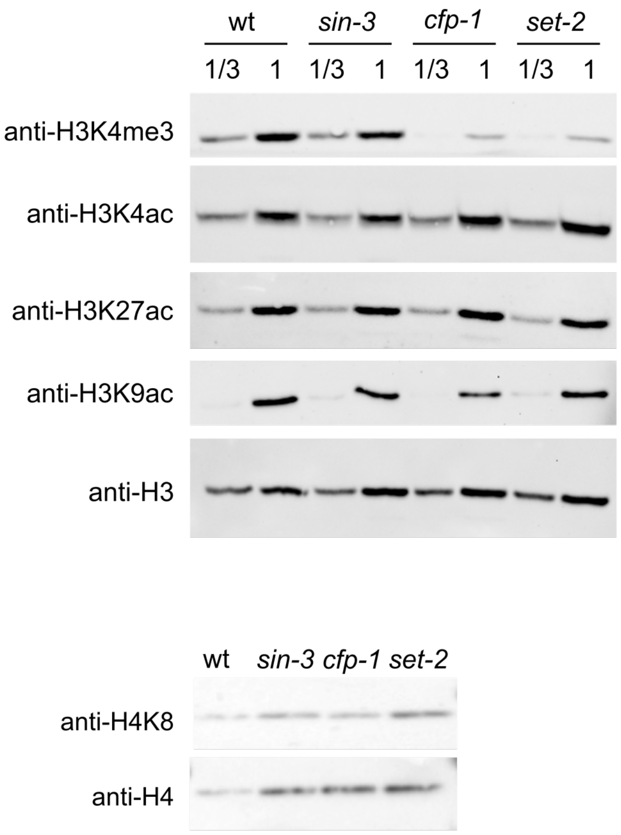
wt
sin-3
cfp-1
set-2
1/3 1 1/3 1 1/3 1 1/3 1
anti-H3K4me3
anti-H3K4ac
anti-H3K27ac
anti-H3K9ac
anti-H3
wt sin-3 cfp-1 set-2
anti-H4K8
anti-H4

B

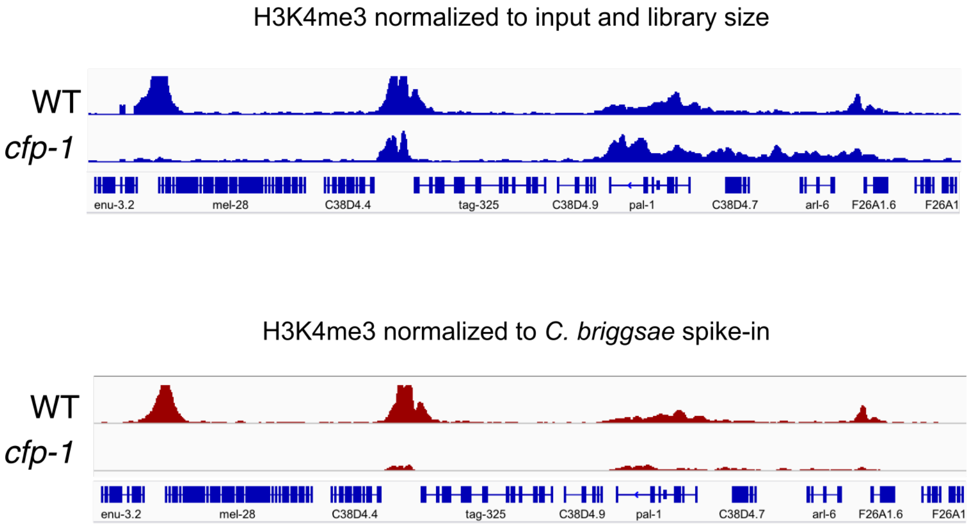
H3K4me3 normalized to input and library size
WT
cfp-1
enu-3.2
mel-28
C38D4.4
tag-325
C38D4.9
pal-1
C38D4.7
arl-6
F26A1.6
F26A1
H3K4me3 normalized to C. briggsae spike-in
WT
cfp-1
enu-3.2
mel-28
C38D4.4
tag-325
C38D4.9
pal-1
C38D4.7
arl-6
F26A1.6
F26A1

Fig. S8

A.

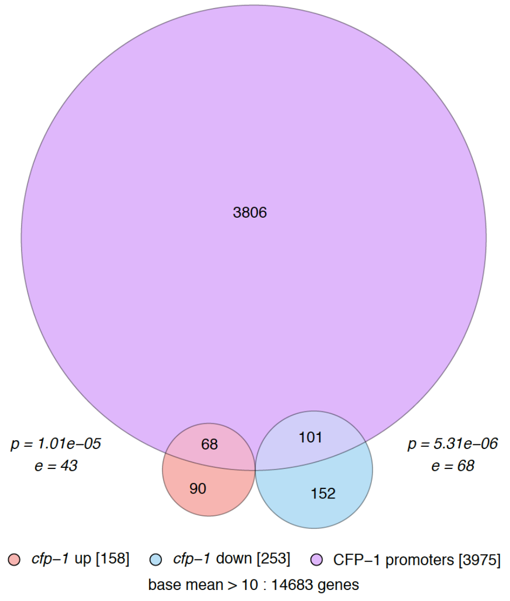
3806
68
101
90
152
p = 1.01e−05
e = 43
p = 5.31e−06
e = 68
cfp−1 up [158]
cfp−1 down [253]
CFP−1 promoters [3975]
base mean > 10 : 14683 genes

B.

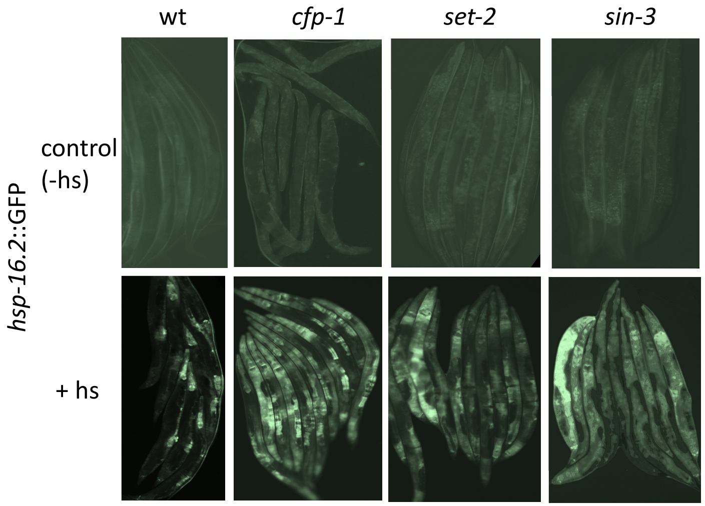
wt
cfp-1
set-2
sin-3
control
(-hs)
+ hs
hsp-16.2::GFP

Fig. S9

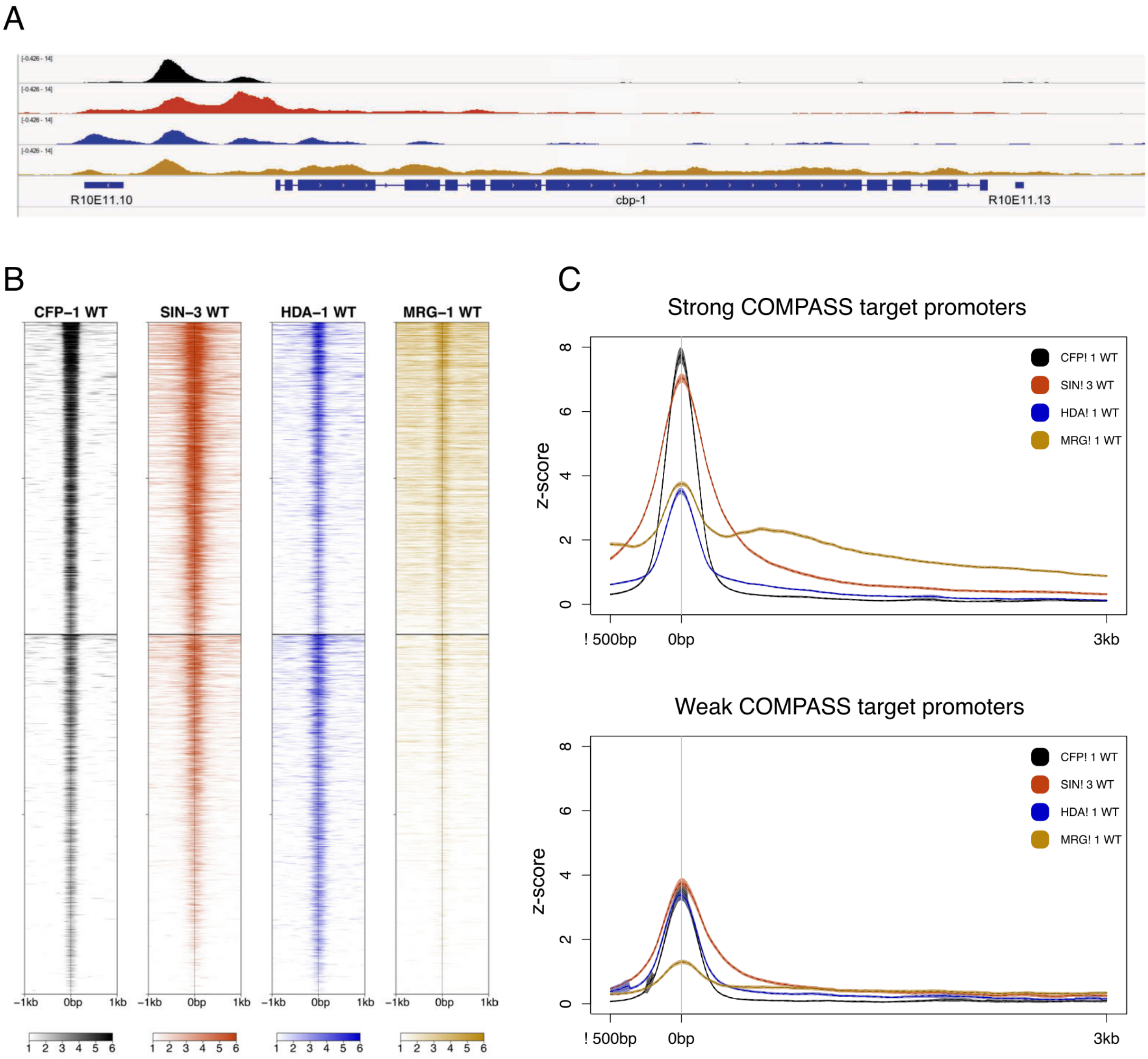
A
R10E11.10
cbp-1
R10E11.13
B
CFP–1 WT
SIN–3 WT
HDA–1 WT
MRG–1 WT
−1kb
0bp
1kb
1 2 3 4 5 6
C
Strong COMPASS target promoters
Weak COMPASS target promoters
z-score
CFP! 1 WT
SIN! 3 WT
HDA! 1 WT
MRG! 1 WT
! 500bp
0bp
3kb

Fig. S10

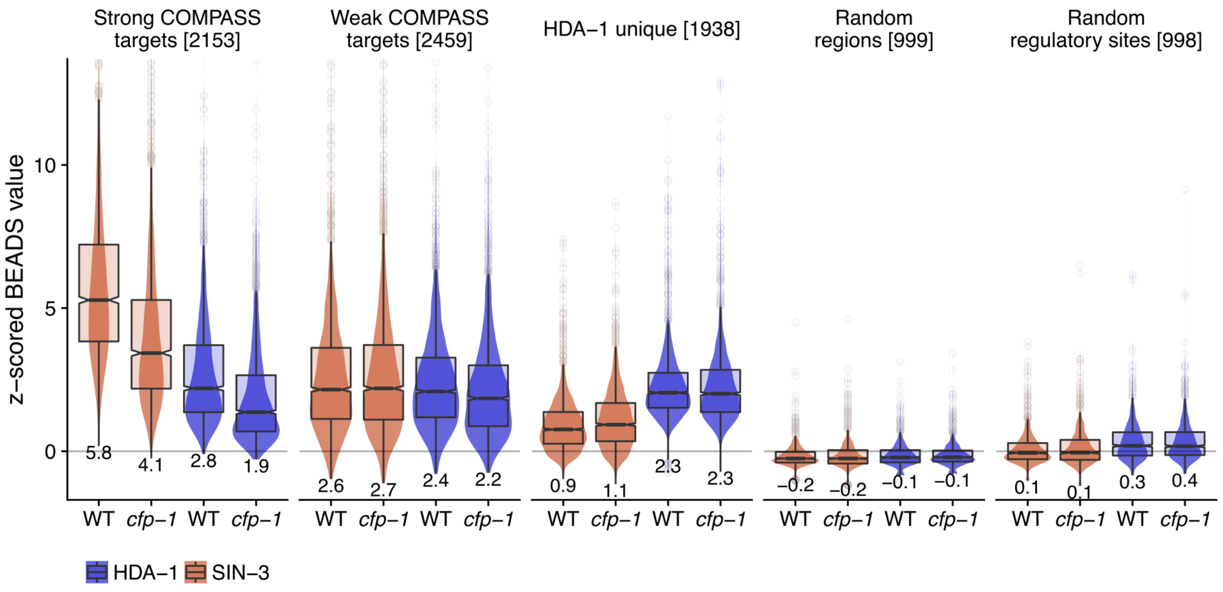

Strong COMPASS targets [2153]
Weak COMPASS targets [2459]
HDA−1 unique [1938]
Random regions [999]
Random regulatory sites [998]
z−scored BEADS value
10
5
0
5.8
4.1
2.8
1.9
2.6
2.7
2.4
2.2
0.9
1.1
2.3
2.3
−0.2
−0.2
−0.1
−0.1
0.1
0.1
0.3
0.4
WT
cfp−1
WT
cfp−1
HDA−1
SIN−3

Fig. S11

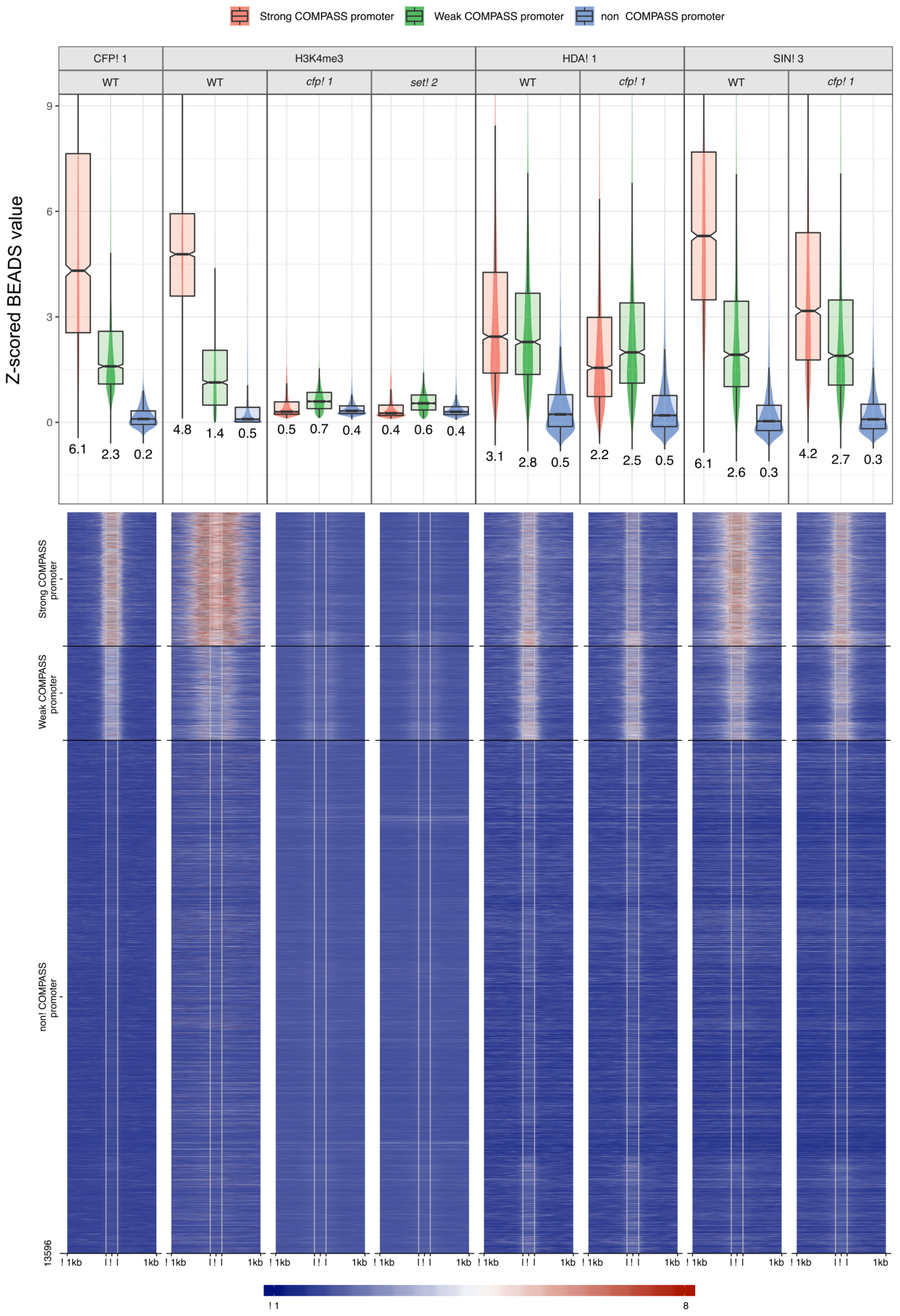

Strong COMPASS promoter
Weak COMPASS promoter
non COMPASS promoter
CFP! 1
H3K4me3
HDA! 1
SIN! 3
WT
cfp! 1
set! 2
Z-scored BEADS value
9
6
3
0
6.1
2.3
0.2
4.8
1.4
0.5
0.5
0.7
0.4
0.4
0.6
0.4
3.1
2.8
0.5
2.2
2.5
0.5
6.1
2.6
0.3
4.2
2.7
0.3
Strong COMPASS promoter
Weak COMPASS promoter
non! COMPASS promoter
13596
! 1kb
1kb
! 1
8
